# Penalized Bayesian forward continuation ratio model with application to high-dimensional data with discrete survival outcomes

**DOI:** 10.1371/journal.pone.0300638

**Published:** 2024-03-28

**Authors:** Anna Eames Seffernick, Kellie J. Archer

**Affiliations:** 1 Department of Biostatistics, St. Jude Children’s Research Hospital, Memphis, TN, United States of America; 2 Division of Biostatistics, College of Public Health, Ohio State University, Columbus, OH, United States of America; Chunghwa Telecom Co. Ltd., TAIWAN

## Abstract

While time-to-event data are often continuous, there are several instances where discrete survival data, which are inherently ordinal, may be available or are more appropriate or useful. Several discrete survival models exist, but the forward continuation ratio model with a complementary log-log link has a survival interpretation and is closely related to the Cox proportional hazards model, despite being an ordinal model. This model has previously been implemented in the high-dimensional setting using the ordinal generalized monotone incremental forward stagewise algorithm. Here, we propose a Bayesian penalized forward continuation ratio model with a complementary log-log link and explore different priors to perform variable selection and regularization. Through simulations, we show that our Bayesian model outperformed the existing frequentist method in terms of variable selection performance, and that a 10% prior inclusion probability performed better than 1% or 50%. We also illustrate our model on a publicly available acute myeloid leukemia dataset to identify genomic features associated with discrete survival. We identified nine features that map to ten unique genes, five of which have been previously associated with leukemia in the literature. In conclusion, our proposed Bayesian model is flexible, allows simultaneous variable selection and uncertainty quantification, and performed well in simulation studies and application to real data.

## Introduction

Often when performing survival analyses, most consider the response to be continuous time-to-event data. However, time-to-event data may also be reported on a discrete, ordinal scale, either for ease of interpretation [1, 2] or because continuous time data are unavailable [3]. For example, the European Society for Medical Oncology (ESMO) clinical recommendations for cutaneous malignant melanoma direct follow-up visits to occur every three months during the first three years and then decrease the frequency to every 6–12 months [3]. Due to this follow-up schedule, time-to-relapse would be an interval censored discrete measurement based on follow-up visit number, as the actual date of relapse is not observed. In another study, researchers classified non-ST-segment elevation myocardial infarction patients as short-, intermediate-, or long-term survivors to investigate the effect of in-hospital major bleeding on time to death [4].

A similar follow-up schedule has been proposed for acute myeloid leukemia (AML), a type of blood cancer. In the 2010 European LeukemiaNet (ELN) guidelines, the recommended follow-up schedule for AML was every 3 months for the first 2 years and then every 6 months up to 5 years [5]. In this case, relapse-free survival would be interval-censored and recorded on a discrete scale. Thus, building models to identify factors associated with these survival outcomes on a discrete scale could provide more clinically meaningful results than using continuous data models. There is particular interest in identifying genomic features related to AML prognosis to further our understanding of the disease. These identified features might be useful prognostic biomarkers and potential targets of novel therapeutic interventions. Many AML datasets are publicly available on repositories such as Gene Expression Omnibus (GEO) including our example dataset, GSE6891 [6, 7].

In low-dimensional settings, many discrete survival models are based on logistic regression, as the censored data likelihood for a discrete survival model can be written in the form of a binary data likelihood [8–10]. However, if we assume the data were generated from a continuous-time proportional hazards model, then we can use a complementary log-log (clog-log) link function to model the covariate dependence of the discrete hazard [11]. Then the estimates from this model are equivalent to those from a Cox proportional hazards model [10]. Hence, this clog-log model is also known as a grouped proportional hazards model [12]. This clog-log model is equivalent to the forward continuation ratio (FCR) ordinal model, and thus has a nice dual survival and ordinal interpretation that is useful for discrete time-to-event outcomes.

To fit a discrete survival model to identify genomic features associated with relapse-free survival, we must take into account the high-dimensional nature of the genomic dataset. The technology used to assay these genomic variables can measure tens of thousands to hundreds of thousands of genomic features for each sample, resulting in the property that the number of features, *P*, exceeds the number of samples, *N*. One way to account for this high-dimensional data is through penalization. Ferber & Archer [13] implemented the penalized clog-log FCR model in the R package ordinalgmifs [14]. This frequentist-based method uses the generalized monotone incremental forward stagewise (GMIFS) algorithm to fit the FCR model with an *ℓ*_1_ penalty. They also incorporate different censoring schemes and the option to include an unpenalized subset of covariates. However, this method has some limitations, namely that it cannot simultaneously perform variable selection and uncertainty quantification and that it is dependent on a single choice of the penalty parameter.

The Bayesian framework can overcome these limitations. Penalized Bayesian models can be used to identify important genomic features while simultaneously performing inference. Additionally, the Bayesian paradigm better accounts for the uncertainty associated with the choice of the penalty parameter by incorporating a prior for this parameter into the hierarchical model.

Herein, we describe a novel Bayesian FCR model which combines the Bayesian Least Absolute Shrinkage and Selection Operator (LASSO) [15–19] with variable inclusion indicators [20–23] to select genomic features that are associated with discrete survival outcomes. In the Materials and methods section, we introduce notation and describe discrete survival models, in particular the FCR model. We also review Bayesian penalization methods, with particular emphasis on the Bayesian LASSO, and present our proposed hierarchical penalized Bayesian FCR model. In the Simulation studies subsection, we describe our simulation design and results. In the Real data application and description subsection, we describe the example AML dataset downloaded from GEO under accession number GSE6891. Our Results and discussion section presents our findings from both our simulation studies and application of our method to the AML dataset. Finally, a brief discussion follows in the Conclusions section.

## Materials and methods

To model these discrete survival responses, we first introduce some notation. Suppose we have *N* independent subjects (*i* = 1, 2, 3, …, *N*), and that each subject has *P* predictors, where *P* > *N*. Thus for each subject a *P* × 1 vector of covariates **x**_*i*_ is observed. Let time be divided into *K* + 1 intervals [*a*_0_ = 0, *a*_1_), [*a*_1_, *a*_2_), …, [*a*_*K*−1_, *a*_*K*_), [*a*_*K*_, *a*_*K*+1_ = ∞) and note that we are assuming time intervals are the same for each subject. Let the discrete survival time response variable be represented by *T*_*i*_ = min(*Y*_*i*_, *C*_*i*_), where *Y*_*i*_ is the event time for subject *i* and *C*_*i*_ is the censoring time of subject *i*. In general, we only observe the minimum of *Y*_*i*_ and *C*_*i*_, not both times. Then *T*_*i*_ = *k* means that the subject experienced the event or was censored in interval [*a*_*k*−1_, *a*_*k*_), also known as time interval *k*, where *k* ∈ {1, …, *K* + 1}.

Define the *N* × 1 vector ***δ*** to be the event indicator, where *δ*_*i*_ = *I*(*Y*_*i*_ < *C*_*i*_). To express the likelihood as in [10, 11, 24], we define an *N* × (*K* + 1) matrix for the event times with elements

$$\begin{array}{c} y_{ik}=\{\begin{array}{cc} 1 & \;\text{if}\;Y_{i}=k\\ 0 & \;\text{otherwise}\; \end{array}. \end{array}$$ (1)

Note that *T*_*i*_ = *Y*_*i*_ when *δ*_*i*_ = 1. That is, uncensored observation times are event times. We begin with the usual censored data likelihood, where censoring is omitted because we assume the parameters do not depend on censored observations [25]

$$\begin{array}{c} L=\prod_{i=1}^{N}\text{Pr}{\left(Y_{i}=k_{i}\right)}^{\delta_{i}}\;\text{Pr}{\left(Y_{i}>k_{i}\right)}^{\left(1-\delta_{i}\right)}. \end{array}$$ (2)

Next we define the discrete hazard rate to be *π*_*ik*_ = *π*_*k*_(**x**_*i*_) = Pr(*Y*_*i*_ = *k*|*Y*_*i*_ ≥ *k*, **x**_*i*_), which is the probability that a subject experiences the event at time interval *k* given that they have not yet experienced the event. The discrete hazard ratio, $\frac{\pi_{ik}}{1-\pi_{ik}}$, is equivalent to a forward continuation ratio in ordinal regression [26]. Using properties of conditional probabilities, we can express the components of the likelihood in Eq (2) as functions of the discrete hazard rate: $\text{Pr}\left(Y_{i}=k_{i}\right)=\pi_{ik_{i}}\prod_{j=1}^{k_{i}-1}\left(1-\pi_{ij}\right)$ and $\text{Pr}\left(Y_{i}>k_{i}\right)=\prod_{j=1}^{k_{i}}\left(1-\pi_{ij}\right),$ where *k*_*i*_ ∈ {1, 2, …, *K* + 1}. Substituting these expressions into Eq (2) yields the following likelihood:

$$\begin{array}{c} L=\prod_{i=1}^{N}{\left[\pi_{ik_{i}}\prod_{j=1}^{k_{i}-1}\left(1-\pi_{ij}\right)\right]}^{\delta_{i}}{\left[\prod_{j=1}^{k_{i}}\left(1-\pi_{ij}\right)\right]}^{\left(1-\delta_{i}\right)}. \end{array}$$ (3)

The equivalent log-likelihood can be written as

$$\begin{array}{c} \text{log}\;L=\sum_{i=1}^{N}\sum_{j=1}^{k_{i}}\;y_{ij}\;\text{log}\;(\frac{\pi_{ij}}{1-\pi_{ij}})+\sum_{i=1}^{N}\sum_{j=1}^{k_{i}}\;\text{log}\left(1-\pi_{ij}\right), \end{array}$$ (4)

because $\sum_{i=1}^{N}\sum_{j=1}^{k_{i}}y_{ij}=\sum_{i=1}^{N}\delta_{i}$. Simplifying further,

$$\begin{array}{c} \text{log}\;L=\sum_{i=1}^{N}\sum_{j=1}^{k_{i}}\left[y_{ij}\;\text{log}\left(\pi_{ij}\right)+\left(1-y_{ij}\right)\;\text{log}\left(1-\pi_{ij}\right)\right]. \end{array}$$ (5)

Notice that Eq (5) is the log-likelihood function for a Bernoulli distribution with response *y*_*ij*_ and probability *π*_*ij*_ [27]. The likelihood in Eq (3) assumes that censoring occurs at the end of the time interval in which censoring was recorded [11]. That is, censored observations are observed at interval *k*_*i*_ but not at interval *k*_*i*_ + 1. However, censoring often occurs in the interior of the survival time interval, and this assumption may lead to bias if the observation was censored soon after the start of interval *k*_*i*_ rather than near the end of the interval [12]. We will assume that censoring occurs at the beginning of the interval in which is recorded, since we assume that once a subject drops out of the study, no additional information is available.

The likelihoods for this alternative censoring scenario can be derived as in [28]. First, consider the case where every subject under study experiences the event of interest. That is, no censoring occurs, as in [1, 29]. The likelihood can be written as the product of *N* conditionally independent binomial random variables. If *π*_*ik*_ = Pr(*Y*_*i*_ = *k*|*Y*_*i*_ ≥ *k*, **x**_*i*_), then 1 − *π*_*ik*_ = Pr(*Y*_*i*_ > *k*|*Y*_*i*_ ≥ *k*, **x**_*i*_). Recall that we previously defined an *N* × (*K* + 1) event matrix, *Y*_*mat*_ which has elements *y*_*ik*_ = 1 if *Y*_*i*_ = *k* and *y*_*ik*_ = 0 otherwise. Then the likelihood under the case of no censoring is given by

$$\begin{array}{c} L=\prod_{i=1}^{N}\prod_{k=1}^{K}\pi_{ik}^{y_{ik}}{\left(1-\pi_{ik}\right)}^{\sum_{j=k}^{K+1}y_{ij}-y_{ik}} \end{array}$$ (6)

Next assume *Y*_*i*_ > *C*_*i*_ − 1. That is, we assume subjects with *C*_*i*_ = *k* are censored at the beginning of interval *k* and so *Y*_*i*_ > *k* − 1. To do this we need to define some additional notation. We define the *N* × (*K* + 1) response matrix, *T*_*mat*_ with elements

$$\begin{array}{c} t_{ik}=\{\begin{array}{cc} 1 & \;\text{if}\;Y_{i}=k\;\text{OR}\;C_{i}=k+1\\ 0 & \;\text{otherwise}\; \end{array} \end{array}$$ (7)

Now the likelihood can be written as

$$\begin{array}{c} L=\prod_{i=1}^{N}\prod_{k=1}^{K+1}\pi_{ik}^{y_{ik}}{\left(1-\pi_{ik}\right)}^{\sum_{j=k}^{K+1}t_{ij}-y_{ik}}. \end{array}$$ (8)

The log-likelihood is given by

$$\begin{array}{c} \text{log}\;\left(L\right)=\sum_{i=1}^{N}\sum_{k=1}^{K+1}[y_{ik}\;\text{log}\left(\pi_{ik}\right)+(\sum_{j=k}^{K+1}\;t_{ij}-y_{ik})\;\text{log}\left(1-\pi_{ik}\right)]. \end{array}$$ (9)

Note that *T*_*i*_ = *C*_*i*_ when *δ*_*i*_ = 0. That is, observation times are equivalent to censoring times for the censored subjects.

We next model the relationship between covariates and the discrete hazard, *π*. Specifically, our application of interest is to identify genomic features that are related to discrete relapse-free survival in AML. Therefore, we are particularly interested in the penalized FCR model with the clog-log link so that we can select features from a high-dimensional set of genomic predictors. The linear form of the FCR model is:

$$\begin{array}{c} \text{log}\left[-\text{log}\left(1-\pi_{ik}\right)\right]=\alpha_{k}+x_{i}\beta \\ k=1,...,K \end{array}$$ (10)

where *α*_*k*_ represents the intercept, or threshold, for the *k*^*th*^ distinct time interval, and ***β*** are the coefficients for the penalized predictors. This model assumes proportional hazards and has the nice property of dual survival and ordinal outcome interpretations. An *ℓ*_1_ penalty can be used to perform variable selection. The penalized solution is:

$$\begin{array}{c} \hat{\beta }=\text{arg}\;\underset{\beta }{\text{max}}\;(\text{log}[L(\alpha ,\beta |y,X)]-\lambda \sum_{m=1}^{P}\left|\beta_{m}\right|) \end{array}$$ (11)

where the tuning parameter λ controls the amount of shrinkage.

The frequentist penalized FCR model proposed by [13] could be used to identify genomic features, but the models are dependent on the choice of the penalty parameter, λ, and do not quantify uncertainty. Discrete random forests might be used to predict survival, but these models are difficult to interpret which is not ideal, as the identified genomic features associated with survival might be useful targets to develop new therapies. To overcome the limitations of these existing methods, we propose a new Bayesian penalized FCR model for high-dimensional data.

Bayesian penalized methods have the advantage of simultaneously performing variable selection and uncertainty quantification, and as well as capturing the variability associated with the choice of the penalty parameters. *ℓ*_1_ penalties, as in the Bayesian LASSO, are very common [16–19]. These models induce this penalization through the use of double-exponential (i.e. Laplace) priors on the regression coefficients. This prior has the form:

$$\begin{array}{c} f\left(\beta_{m}\right)=\frac{1}{2\tau }\;\text{exp}\;(-\frac{|\beta_{m}|}{\tau }) \end{array}$$ (12)

where *τ* = 1/λ and *m* = 1, …, *P*.

To improve variable selection performance, we can multiply the regression coefficients in the Bayesian model by a binary variable *γ* [20–23]. Note that *γ* is a variable inclusion indicator and is given a prior to incorporate a priori information about the probability a variable will be selected into the model. Our group has previously implemented Bayesian LASSO models with variable inclusion indicators for variable selection with ordinal responses [30–33]. Here we extend this idea to the discrete survival FCR model.

### Proposed hierarchical penalized Bayesian forward continuation ratio model

Our hierarchical Bayesian LASSO FCR model with clog-log link and variable inclusion indicators is given by:

$$\begin{array}{cc} \pi_{ik}\left(x_{i}\right) & =1-\text{exp}\{-\text{exp}(\alpha_{k}+X_{i}D_{\gamma }\beta )\}\\ D_{\gamma } & =\text{diag}(\gamma_{1},...,\gamma_{P})\\ \alpha_{k} & ∼\text{Normal}(0,\sigma^{2})\;\text{for}\;k=1,...,K\\ \beta_{m}|\lambda & ∼\text{LaPlace}(0,1/\lambda )\\ \lambda & ∼\text{Gamma}(a,b)\\ \gamma_{m} & ∼\text{Binomial}(1,\theta_{m}) \end{array}$$ (13)

We will set *θ*_*m*_ = *s* for some fixed *s*.

We will use the log-likelihood in Eq (9) together with the hierarchical model in Eq (13) to define the posterior distribution. When performing the posterior sampling, we use the event matrix, *Y*_*mat*_, with elements given by Eq (1) and the response matrix, *T*_*mat*_, with elements given by Eq (7). We implement the Bernoulli form of the model by restructuring *Y*_*mat*_ and the cumulative response matrix, *T*_*cum*_, into long format and removing cases where cumulative *T* is zero. A matrix in wide format can be converted into long format by stacking the rows of the matrix into columns and labeling the entries with the appropriate column names. For example, let our wide matrix have the form

$$\begin{array}{c} \begin{array}{l} \;\;\;Y_{1}\;Y_{2}\;\;Y_{3} \end{array}\\ \left(\begin{array}{ccc} 1 & 0 & 0\\ 0 & 0 & 1\\ 0 & 1 & 0 \end{array}\right) \end{array}$$

Then the long format of this matrix is

$$\begin{array}{l} \begin{array}{cc} \;\;\;Y & Indicatior \end{array}\\ \left(\begin{array}{cccccc} Y_{1} & & & 1 & & \\ Y_{1} & & & 0 & & \\ Y_{1} & & & 0 & & \\ Y_{2} & & & 0 & & \\ Y_{2} & & & 0 & & \\ Y_{2} & & & 1 & & \\ Y_{3} & & & 0 & & \\ Y_{3} & & & 1 & & \\ Y_{3} & & & 0 & & \end{array}\right) \end{array}$$

Proof of unimodality of the posterior of ***β*** under hierarchical model in Eq 13 is provided in S1 Appendix.

### Variable selection with variable inclusion indicators

Though the Bayesian LASSO can perform automatic variable selection when using posterior models, it is more common to summarize posterior distributions using means or medians. Often posterior intervals, such as credible intervals (CI) or highest posterior density intervals (HPDI) are used to perform variable selection. A variable is selected into the model if its CI or HPDI does not contain zero. Specific variable selection methods related to variable inclusion indicators have also been developed. One option is to use the posterior probabilities of the inclusion indicators (Pr(*γ*|*Data*)) as in [20, 22, 34]. A variable is selected into the model if it’s associated variable inclusion indicator, *γ*, has a posterior probability greater than 0.5.

Another variable selection method for variable inclusion indicators is to use Bayes factors. The Bayes factor (BF) is defined as the ratio of posterior odds to prior odds, where

$$\begin{array}{c} \text{Prior}\;\text{Odds}=\frac{\text{Pr}(H_{A})}{\text{Pr}(H_{0})}, \end{array}$$ (14)

$$\begin{array}{c} \text{Posterior}\;\text{Odds}=\frac{\text{Pr}(H_{A}|Data)}{\text{Pr}(H_{0}|Data)}, \end{array}$$ (15)

and

$$\begin{array}{c} BF=\frac{\text{Posterior}\;\text{Odds}}{\text{Prior}\;\text{Odds}}. \end{array}$$ (16)

To apply Bayes factors to *β*, we test *H*_0_ : |*β*| ≤ *ϵ* vs. *H*_*A*_ : |*β*| > *ϵ*, as in [35]. Using the marginal prior for *β* derived in S1 Appendix and assuming that *ϵ* is a small positive value close to 0, we have

$$\begin{array}{cc} \text{Pr}(|\beta |\leq \epsilon ) & =\text{Pr}(-\epsilon \leq \beta <0)+\text{Pr}(0\leq \beta \leq \epsilon )\\ & =\int_{-\epsilon }^{0}\frac{b^{a}\Gamma \left(a+1\right)}{2\Gamma (a)}\frac{1}{{\left(b-\beta \right)}^{a+1}}d\beta +\int_{0}^{\epsilon }\frac{b^{a}\Gamma \left(a+1\right)}{2\Gamma (a)}\frac{1}{{\left(b+\beta \right)}^{a+1}}d\beta \\ & =\frac{b^{a}\Gamma (a+1}{a\Gamma (a)}[\frac{1}{b^{a}}-\frac{1}{{\left(b+\epsilon \right)}^{a}}] \end{array}$$

$$\begin{array}{cc} \text{Pr}(|\beta |>\epsilon ) & =\text{Pr}(\beta <-\epsilon )+\text{Pr}(\beta >\epsilon )\\ & =\int_{-\infty }^{-\epsilon }\frac{b^{a}\Gamma \left(a+1\right)}{2\Gamma (a)}\frac{1}{{\left(b-\beta \right)}^{a+1}}d\beta +\int_{\epsilon }^{\infty }\frac{b^{a}\Gamma \left(a+1\right)}{2\Gamma (a)}\frac{1}{{\left(b+\beta \right)}^{a+1}}d\beta \\ & =\frac{b^{a}\Gamma \left(a+1\right)}{a\Gamma (a)}\frac{1}{{\left(b+\epsilon \right)}^{a}} \end{array}$$

The prior odds can therefore be derived as

$$\frac{\text{Pr}(|\beta |>\epsilon )}{\text{Pr}(|\beta |\leq \epsilon )}=\frac{b^{a}}{{\left(b+\epsilon \right)}^{a}}.$$

The posterior odds will be obtained using the posterior samples.

Similarly, to apply Bayes factor methodology to *βγ* we test the hypothesis *H*_0_ : |*βγ*| ≤ *ϵ* vs. *H*_*A*_ : |*βγ*| > *ϵ* for some small value *ϵ*. Under the hierarchical model, assuming *ϵ* > 0 and λ and *β* are independent,

$$\begin{array}{cc} \text{Pr}(|\gamma \beta |\leq \epsilon ) & =\text{Pr}(-\epsilon \leq \gamma \beta \leq \epsilon )\\ & =\text{Pr}(\gamma \beta =0)+\text{Pr}(-\epsilon \leq \gamma \beta <0)+\text{Pr}(0<\gamma \beta \leq \epsilon )\\ & =\text{Pr}(\gamma =0)+\text{Pr}(\gamma =1)\text{Pr}(-\epsilon \leq \beta <0)+\text{Pr}(\gamma =1)\text{Pr}(0<\beta \leq \epsilon )\\ & =\text{Pr}\left(\gamma =0\right)+\text{Pr}\left(\gamma =1\right)\int_{-\epsilon }^{0}\frac{b^{a}\Gamma \left(a+1\right)}{2\Gamma (a)}\frac{1}{{\left(b-\beta \right)}^{a+1}}d\beta \\ & \;+\text{Pr}\left(\gamma =1\right)\int_{0}^{\epsilon }\frac{b^{a}\Gamma \left(a+1\right)}{2\Gamma (a)}\frac{1}{{\left(b+\beta \right)}^{a+1}}d\beta \\ & =\text{Pr}\left(\gamma =0\right)+\text{Pr}\left(\gamma =1\right)\frac{b^{a}\Gamma \left(a+1\right)}{a\Gamma (a)}[\frac{1}{b^{a}}-\frac{1}{{\left(b+\epsilon \right)}^{a}}], \end{array}$$

$$\begin{array}{cc} \text{Pr}(|\gamma \beta |>\epsilon ) & =\text{Pr}(\gamma \beta >\epsilon )+\text{Pr}(\gamma \beta <-\epsilon )\\ & =\text{Pr}(\gamma =1)\text{Pr}(\beta >\epsilon )+\text{Pr}(\gamma =1)\text{Pr}(\beta <-\epsilon )\\ & =\text{Pr}\left(\gamma =1\right)\int_{\epsilon }^{\infty }\frac{b^{a}\Gamma \left(a+1\right)}{2\Gamma (a)}\frac{1}{{\left(b+\beta \right)}^{a+1}}d\beta \\ & +\text{Pr}\left(\gamma =1\right)\int_{-\infty }^{-\epsilon }\frac{b^{a}\Gamma \left(a+1\right)}{2\Gamma (a)}\frac{1}{{\left(b-\beta \right)}^{a+1}}d\beta \\ & =\text{Pr}\left(\gamma =1\right)\frac{b^{a}\Gamma \left(a+1\right)}{a\Gamma (a)}\frac{1}{{\left(b+\epsilon \right)}^{a}} \end{array}$$

The prior odds can therefore be derived as

$$\frac{\text{Pr}(|\gamma \beta |>\epsilon )}{\text{Pr}(|\gamma \beta |\leq \epsilon )}=\frac{\text{Pr}\left(\gamma =1\right)b^{a}\Gamma \left(a+1\right)}{\text{Pr}\left(\gamma =1\right)\left({\left(b+\epsilon \right)}^{a}-b^{a}\right)\Gamma \left(a+1\right)+\text{Pr}\left(\gamma =0\right)a{\left(b+\epsilon \right)}^{a}\Gamma \left(a\right)}$$

and the posterior odds will be calculated using the posterior samples.

Finally, we can also apply the BF methodology directly to the inclusion indicator *γ*. When applying the Bayes factor to *γ*, we test the hypothesis *H*_0_ : *γ* = 0 vs. *H*_*A*_ : *γ* = 1. The prior odds depend on the choice of hyper parameter on *θ*. When *θ*_*m*_ = *s* then *γ*_*m*_ ∼ *Bin*(1, *s*) and so the prior odds are

$$\begin{array}{c} \frac{\text{Pr}(H_{A})}{\text{Pr}(H_{0})}=\frac{\text{Pr}(\gamma =1)}{\text{Pr}(\gamma =0)}=\frac{s}{1-s}. \end{array}$$ (17)

The posterior odds will be found empirically from the posterior samples. Typically with BF methodology, a threshold is chosen to determine variable importance [36]. We will consider genomic features important if their BF exceeds 5.

### Simulation studies

Simulation studies for the high-dimensional Bayesian penalized FCR models were conducted in R version 4.1.2 [37]. Bayesian analysis was performed using Just Another Gibbs Sampler (JAGS) [38] with the runjags R package [39]. We utilized the high performance computing power of the Ohio Supercomputer Center (OSC) [40].

#### Simulation design

We used an existing dataset to generate our simulated data, so as to better capture the complex correlation structure present in high-dimensional genomic datasets. To generate simulated data, we used a publicly available gene expression dataset of AML patients in the German AMLCG 1999 trial (GEO accession number: GSE37642) [41–44]. These publicly available archived data are fully de-identified and thus are not considered human subjects research. The data were read into R using the GEOquery library. This dataset contains 562 samples with gene expression measured using different Affymetrix arrays (140 HGU-133plus2; 422 HGU-133A; 422 HGU-133B). We used only the U133A GeneChip data in our simulation. After excluding patients that were missing overall survival and removing the control probesets, we had 417 samples and 22215 features for our simulations and the overall censoring rate was 26.1%. The time-interval-specific censoring rate is reported in Table 1, where the interval cutpoints were chosen so that the sample size was balanced across the intervals.

**Table 1:** Censoring rates for each time interval in the GSE37642 dataset.

Time-interval	Proportion Censored
1	0.000
2	0.061
3	0.059
4	0.301
5	0.881

Before building our simulation datasets, we preprocessed these data. We used the caret package to filter the data to remove probesets with near zero variance and probesets that are highly correlated (*ρ* > 0.75) [45]. Then we applied a variance filter to keep the top 1,000 most variable probesets. We also centered and scaled the expression values prior to generating the survival outcomes.

We generated both balanced and unbalanced simulation datasets. For both settings, we generated survival data by first selecting five features in the dataset to have *β* = log(2), an additional five features to have *β* = −log(2), and the remaining 990 features to have *β* = 0. We then used this ***β*** vector to generate the linear predictor, and next randomly generated survival times with rate equal to exp(***β***^*T*^**X**). We randomly generated censoring time from an exponential distribution with rate equal to 0.2676, which was the estimated rate from an intercept-only exponential survival model fit to the data. We set the observed time to be the minimum of the event time and the censoring time and created the censoring indicator. Finally, we grouped the continuous survival times into 5 discrete intervals.

For the balanced simulation datasets, we used the quintiles of the first simulated dataset to define the grouping, so that there would be a robust sample size for model fitting in each time interval. The thresholds were 0.09, 0.32, 0.67 and 1.7. Summary statistics of the quintiles across the 100 simulated datasets are reported in Table 2 and are on average close to the thresholds used. The mean proportion of samples per interval and the mean censoring proportion for the 100 simulated balanced datasets are reported in Table 3. The sample size is similar in the five intervals, and the censoring rate increases over time (as the interval number increases), as in the actual dataset.

**Table 2:** Summary statistics of quintiles across the 100 simulated balanced datasets generated using the GSE37642 dataset.

Percentile	Min	1^st^ Quartile	Median	Mean	3^rd^ Quartile	Max
20%	0.06	0.07	0.08	0.08	0.09	0.11
40%	0.20	0.25	0.26	0.27	0.29	0.34
60%	0.57	0.65	0.69	0.69	0.73	0.82
80%	1.37	1.63	1.72	1.73	1.84	2.14

**Table 3:** Average proportion of samples per time-interval and average interval-specific censoring proportion across the 100 simulated balanced datasets generated using the GSE37642 dataset.

Time Interval	Proportion of Samples	Proportion Censored
1	0.218	0.096
2	0.217	0.187
3	0.158	0.291
4	0.203	0.393
5	0.204	0.574

For the unbalanced simulation datasets, we defined the intervals to be similar to those in the ELN guidelines [5]. The thresholds were 0.25, 0.5, 0.75, and 1 to reflect the recommended follow-up schedule of every 3 months for the first year. The mean proportion of samples per interval and the mean censoring proportion for the 100 simulated unbalanced datasets are reported in Table 4. Now there are more samples in the first and last classes, which reflects the actual AML dataset. The censoring rate increases over time, as in the actual dataset and the simulated unbalanced datasets.

**Table 4:** Average proportion of samples per time-interval and average interval-specific censoring proportion across the 100 simulated unbalanced datasets generated using the GSE37642 dataset.

Time Interval	Proportion of Samples	Proportion Censored
1	0.386	0.132
2	0.144	0.253
3	0.091	0.311
4	0.065	0.374
5	0.314	0.519

For each dataset, we fit our proposed Bayesian LASSO FCR model with variable inclusion indicators (BLASSO-FCR) in Eq (13) with *s* = 0.01, 0.1, or 0.5. These models were fit using runjags on OSC with 500 adaptation steps and 500 burn-in steps, which were discarded. We then fit 3 chains and thinned every third step so that we saved a total of 9,999 iterations. We assessed model convergence using potential scale reduction factor (PSRF), where *PSRF* > 1.1 indicates lack of convergence [46]. Sample R code can be found in S1 Appendix. Code for a toy example can be found at https://github.com/annaSeffernick/BayesianLassoFCR. We also fit the frequentist OGMIFS FCR model for comparison.

In the simulation studies, variable selection performance was assessed using true positive rates (TPR), true negative rates (TNR), positive predictive values (PPV), negative predictive values (NPV), and false discovery rates. The definition of these quantities are as follows:

$$TPR=\frac{\#\;\text{True}\;\text{Positives}\;\text{Identified}}{\#\;\text{True}\;\text{Positives}},$$

$$TNR=\frac{\#\;\text{True}\;\text{Negatives}\;\text{Identified}}{\#\;\text{True}\;\text{Negatives}},$$

$$PPV=\frac{\#\;\text{True}\;\text{Positives}\;\text{Identified}}{\#\;\text{Discoveries}\;\text{Identified}},$$

$$NPV=\frac{\#\;\text{True}\;\text{Negatives}\;\text{Identified}}{\#\;\text{Negatives}\;\text{Identified}},$$

and

$$FDR=\frac{\#\;\text{False}\;\text{Discoveries}\;\text{Identified}}{\#\;\text{Discoveries}\;\text{Identified}}.$$

Ideally, TPR, TNR, PPV, and NPV will be 1 and FDR will be close to 0. Note that we are not strictly controlling FDR. These quantities will be calculated for the different variable selection methods described in the Methods Section. For the BLASSO-FCR models, we use Bayes factors (BF) for *β*, *γβ*, and *γ*, posterior probabilities (Pr(*γ*|*D*) > 0.5), and 95% credible intervals (CI). For the OGMIFS models, features are selected using Akaike Information Criterion (AIC) and Bayesian Information Criterion (BIC).

### Real data application and description

To identify genomic features predictive of discrete survival, we applied our proposed BLASSO-FCR model to a publicly available gene expression dataset (GEO accession number: GSE6891) [6, 7]. These publicly available archived data are fully de-identified and thus are not considered human subjects research. This dataset includes 521 AML patients (≤ 60 years old), was collected using Affymetrix HG U133 Plus 2.0 GeneChip arrays, and the outcome of interest was relapse-free survival (RFS). We grouped RFS into 5 discrete time intervals based on 4 cutpoints: 6 months, 12 months, 18 months, and 24 months, as described in the ELN guidelines [5]. We used cutpoints every six months rather than 3 months so that each group had large enough sample size for robust estimation. The distribution of samples across these 5 time intervals is presented in Table 5.

**Table 5:** Distribution of GSE6891 patients across relapse-free survival groups. Time interval 1 is defined as 0 to 6 months, interval 2 is 6 to 12 months, interval 3 is 12 to 18 months, interval 4 is 18 to 24 months, and interval 5 is greater than 24 months.

Time Interval	Censored	Relapsed	Total
1	0	65	65
2	2	107	109
3	0	43	43
4	0	14	14
5	138	45	183

Prior to applying our Bayesian method, we processed the data through a number of filtering steps. The data were read into R using the GEOquery library [47]. Initially, there were 521 samples and 54,675 genomic features. We first removed 107 samples who were missing RFS. Next we removed genomic features that had missing values (*p* = 62). Then we used the nzv function in the caret package to identify features with near zero variance [45]. While none of the features were flagged as having zero variance, there were several features with the majority of samples having the same expression values. Thus, we filtered to keep only those features with at least 20% unique values, based on the 1st quartile cutoff of the percentage of unique values across all features. This left 40,636 features. We additionally used the caret package to remove highly correlated features (*ρ* > 0.75), which left 33,011. Next, we applied a variance filter to keep the 1,000 most variable features. Finally, we centered and scaled the expression values before fitting the Bayesian models. The final application dataset contains 414 samples and 1000 gene expression variables.

As in the simulation studies, we fit our proposed BLASSO-FCR model with *θ* = 0.01, 0.1, 0.5 and evaluated the two best performing BF variable selection methods. All analyses were done in R version 4.1.2. [37] Bayesian models were fit using JAGS with the runjags R package [39] on OSC [40].

## Results and discussion

### Simulation results

**Fig 1:**
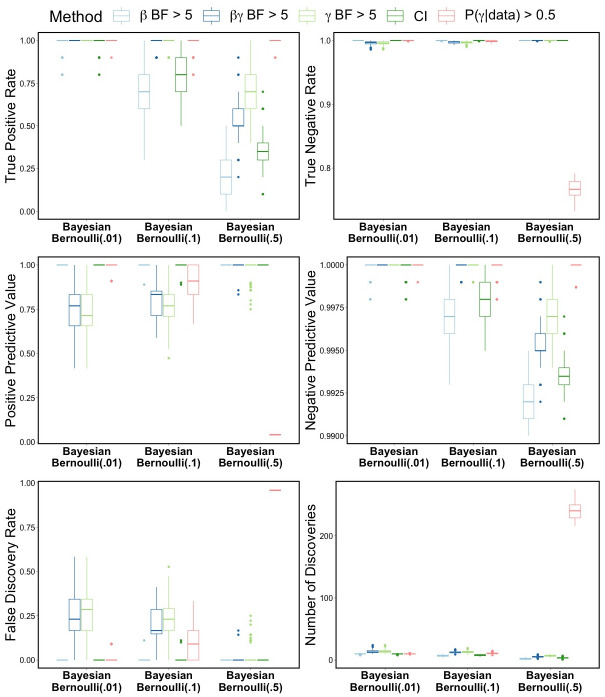
Balanced data simulation results. Variable selection performance for Bayesian LASSO FCR model fit to simulated balanced data containing 10 truly related features among 1000 covariates from GSE37642 dataset.

The variable selection results for the BLASSO-FCR models are reported in Table 6 and Fig 1 for the simulated balanced datasets. The *βγ* and *γ* BF methods tend to perform quite well. PPVs and FDRs are improved for larger values of *θ*, while TPRs and NPVs worsen slightly as *θ* increases. The CI method also has good performance, but the *βγ* BF and *γ* BF methods generally perform the best in terms of TPR and NPV, and these methods select close to the true number of features. The Pr(*γ*|*D*) selection method for *θ* = 0.5 identifies a very large number of features which decreases the TNR and PPV and increases FDR. For TPR, TNR, and NPV, the oracle value *θ* = 0.01 performs best.

**Table 6:** Variable Selection performance from Bayesian LASSO FCR model with different prior inclusion probabilities *θ* fit to simulated balanced data containing 10 significant features with 1000 covariates from GSE37642 dataset. Model was selected using credible intervals (CI), Bayes factors (BF), or mean posterior probability of inclusion (Pr(*γ*|*D*)).

*θ*	Method	Discoveries	FDR	TPR	TNR	PPV	NPV
0.01	CI	9.86	0	0.986	1	1	0.9999
	*βγ* BF > 5	13.82	0.247	1	0.996	0.753	1
	*β* BF > 5	9.87	0	0.987	1	1	0.9999
	*γ* BF > 5	13.85	0.249	1	0.996	0.751	1
	Pr(*γ*|*D*) > 0.5	10.07	0.007	0.999	0.9999	0.993	0.99999
0.1	CI	8.04	0.002	0.802	0.99998	0.998	0.998
	*βγ* BF > 5	12.55	0.200	0.989	0.997	0.800	0.9999
	*β* BF > 5	7.11	0.001	0.710	0.99999	0.999	0.997
	*γ* BF > 5	13.1	0.231	0.989	0.997	0.769	0.9999
	Pr(*γ*|*D*) > 0.5	10.85	0.0939	0.976	0.999	0.907	0.9998
0.5	CI	3.53	0	0.353	1	1	0.994
	*βγ* BF > 5	5.43	0.003	0.541	0.99998	0.997	0.995
	*β* BF > 5	1.98	0	0.198	1	1	0.992
	*γ* BF > 5	7.17	0.032	0.691	0.9997	0.968	0.997
	Pr(*γ*|*D*) > 0.5	240.11	0.958	0.997	0.768	0.042	0.99996

**Fig 2:**
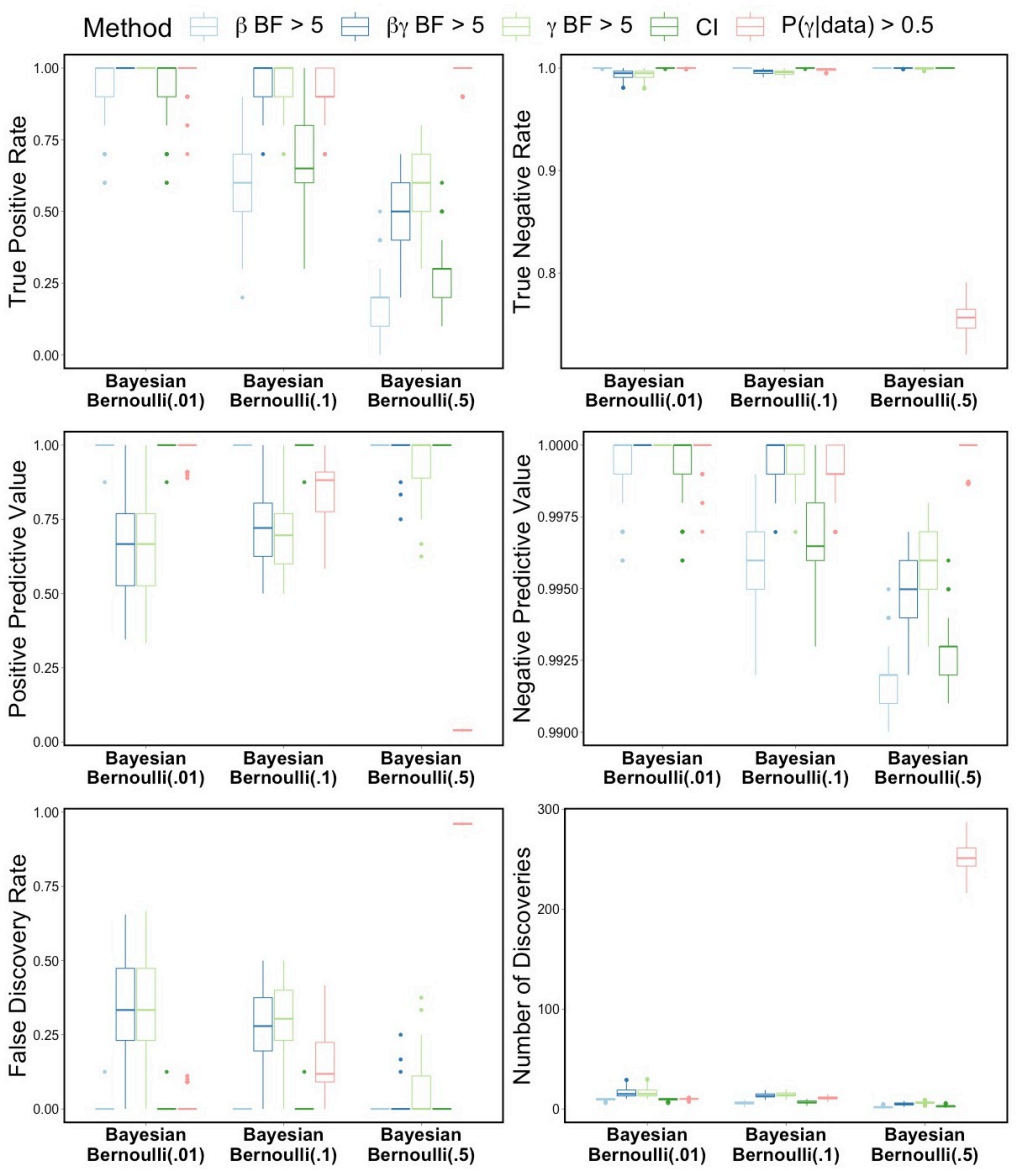
Unbalanced data simulation results. Variable selection performance for Bayesian LASSO FCR model fit to simulated unbalanced data containing 10 truly related features among 1000 covariates from GSE37642 dataset.

The variable selection results from the BLASSO-FCR model fit for the simulated unbalanced datasets are in Table 7 and Fig 2. The results are very similar to those for the balanced datasets, with very similar patterns across the values of *θ*. The positive predictive values are slightly lower and the FDR and number of discoveries slightly higher for these unbalanced datasets compared to the balanced datasets.

**Table 7:** Variable Selection performance from Bayesian LASSO FCR model with different prior inclusion probabilities *θ* fit to simulated unbalanced data containing 10 significant features with 1000 covariates from GSE37642 dataset. Model was selected using credible intervals (CI), Bayes factors (BF), or mean posterior probability of inclusion (Pr(*γ*|*D*)).

*θ*	Method	Discoveries	FDR	TPR	TNR	PPV	NPV
0.01	CI	9.44	0.00125	0.943	0.99999	0.999	0.9994
	*βγ* BF > 5	16.25	0.347	1	0.994	0.653	1
	*β* BF > 5	9.41	0.00125	0.94	0.99999	0.999	0.9994
	*γ* BF > 5	16.25	0.3467	1	0.994	0.653	1
	Pr(*γ*|*D*) > 0.5	9.98	0.011	0.987	0.9999	0.989	0.9999
0.1	CI	6.63	0.00125	0.662	0.99999	0.999	0.997
	*βγ* BF > 5	13.52	0.276	0.957	0.996	0.724	0.9996
	*β* BF > 5	5.74	0	0.574	1	1	0.996
	*γ* BF > 5	14.25	0.309	0.962	0.995	0.691	0.9996
	Pr(*γ*|*D*) > 0.5	10.86	0.146	0.916	0.998	0.854	0.999
0.5	CI	2.67	0	0.267	1	1	0.993
	*βγ* BF > 5	4.87	0.005	0.484	0.99997	0.995	0.995
	*β* BF > 5	1.71	0	0.171	1	1	0.992
	*γ* BF > 5	6.54	0.044	0.62	0.9997	0.956	0.996
	Pr(*γ*|*D*) > 0.5	251.89	0.961	0.989	0.756	0.039	0.9999

The convergence results for both simulation settings are in Table 8. As the fixed value of *θ* decreased, the number of datasets with lack of convergence increased and the mean number of parameters with *PSRF* > 1.1 out of the 3016 monitored parameters also increased. Convergence was especially poor for the oracle value *θ* = 0.01, with all datasets having at least one parameter that failed to converge and a much higher mean number of parameters with *PSRF* > 1.1. For *θ* = 0.1 and *θ* = 0.5, only a small number of parameters failed to converge.

**Table 8:** Lack of convergence for BLASSO-FCR model fit to GSE37642 simulated balanced and unbalanced data with *θ* = 0.01, 0.1, 0.5 in terms of *PSRF* > 1.1. The third column corresponds to the number of datasets out of 100 simulated datasets in which at least one parameter failed to converge. The fourth column corresponds to the average number of parameters out of 3016 monitored parameters that had *PSRF* > 1.1.

Setting	*θ*	Number of Datasets	Mean Number of Parameters
Balanced	0.01	100	153.87
	0.1	91	1.95
	0.5	48	0.56
Unbalanced	0.01	100	129.36
	0.1	81	1.54
	0.5	35	0.39

**Fig 3:**
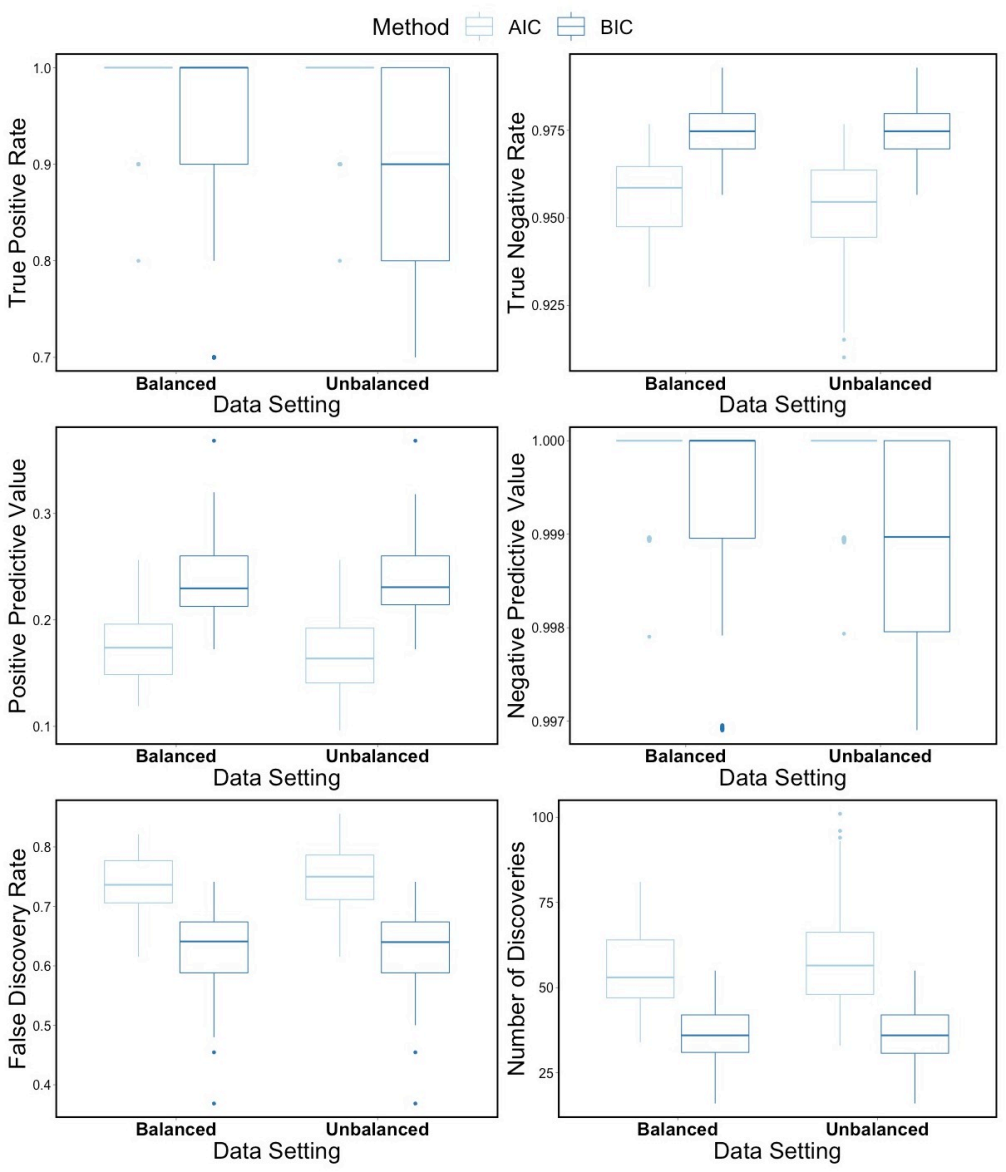
OGMIFS simulation results. Variable selection performance for OGMIFS FCR models fit to 100 balanced and 100 unbalanced simulated datasets containing 10 truly related features among 1000 covariates from GSE37642 dataset.

The OGMIFS FCR model variable selection results are shown in Table 9 and Fig 3. For both balanced and unbalanced simulated data, a large number of features are selected by both AIC and BIC, which leads to high FDRs. As shown in Fig 3, there is little difference in the variable selection metrics across the two simulated data settings. These models perform well in terms of TPR, NPV, and NPV. However, the PPVs are pretty low, generally below 0.3. For TNR, PPV, and FDR the BIC selection method performs better than the AIC selection method. However, the AIC method has higher TPR and NPV compared to the BIC method.

**Table 9:** Variable Selection performance from OGMIFS FCR model with optimal step selected with AIC or BIC, fit to simulated balanced and unbalanced data containing 10 truly related features among 1000 covariates from GSE37642 dataset.

Setting	Method	Discoveries	FDR	TPR	TNR	PPV	NPV
Balanced	AIC	54.87	0.735	0.984	0.957	0.176	0.9998
	BIC	36.38	0.629	0.917	0.975	0.239	0.999
Unbalanced	AIC	58.46	0.747	0.981	0.953	0.168	0.9998
	BIC	36.1	0.628	0.911	0.975	0.239	0.999

**Fig 4:**
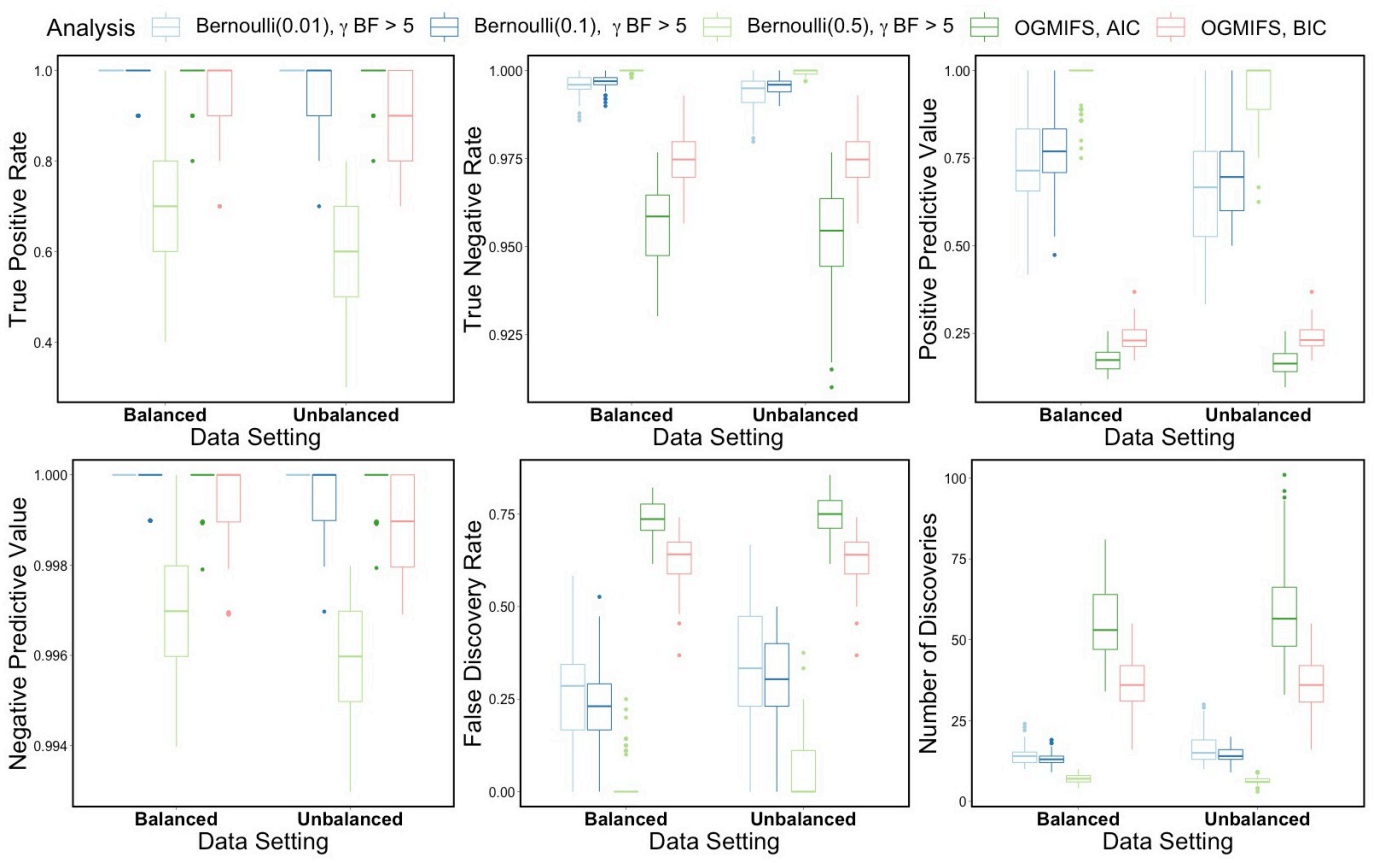
Simulation comparison of BLASSO-FCR and OGMIFS FCR models. Comparison of variable selection performance for BLASSO-FCR models with features selected using *γ* BF > 5 and the OGMIFS FCR model with optimal step selected using AIC or BIC, fit to 100 balanced and 100 unbalanced simulated datasets with 10 significant features and covariates from the GSE37642 dataset.

We also compared the variable selection performance of the BLASSO-FCR model with *γ* BF selection to the OGMIFS methods across values of *θ* and censoring scenarios. These results are shown in Fig 4 and similar patterns are seen for the balanced and unbalanced simulated datasets across the variable selection metrics. For TPR and NPV, all methods perform very well, except for the Bayesian model with *θ* = 0.5. The OGMIFS model with BIC selection also has slightly lower TPR and NPV rates but not as low as the BLASSO-FCR model with *θ* = 0.5. For TNR and PPV, the Bayesian models have much higher values than the OGMIFS models. The Bayesian methods have lower FDR compared to the OGMIFS methods for censoring scenarios both simulation data settings. Finally, the OGMIFS models made more discoveries than the Bayesian models for all censoring scenarios.

These simulation results show that in general, the Bayesian methods outperform the OGMIFS methods. Among the Bayesian methods, the variable selection performance is very similar across the simulated data settings. Among the variable selection methods, *βγ* and *γ* BF methods performed best. Due to its straightforward implementation, we recommend using *γ* BF for variable selection in practice. Based on convergence results, it seems that a choice of *θ* = 0.1 for prior inclusion probability performed well and might be useful in practice. However, this is data-dependent and should be explored for each application.

We performed additional simulations to explore the performance of the proposed BLASSO-FCR model when the generated data had smaller sample sizes, a larger number of truly important features, and a higher censoring rate. These results can be found in S1 Appendix. These results show that variable selection performance generally improves as the sample size increases, that there is little difference between the original censoring level (26%) and the increased censoring level (40%), and that the selection performance is sensitive to the choice of *θ* as well as the selection method. When applying the BLASSO-FCR model to real data, care must be taken when setting *θ* and in choosing the BF threshold. One could also decide to focus on the features with the largest BF, for example, ordering the genomic features by BF and selecting the top 100 or 500 features. It is also important to evaluate convergence and increase the number of MCMC iterations until all parameters converge.

### Application results

We first assessed the convergence of our proposed models under the different *θ* values, reported in Table 10. As in our simulations, convergence was generally quite good for these models. There was only a small number of monitored parameters with PSRF > 1.1 for *θ* = 0.01 and no evidence of lack of convergence for *θ* = 0.1 and *θ* = 0.5.

**Table 10:** Lack of convergence (PSRF > 1.1) for BLASSO-FCR model fit with *θ* = 0.01, 0.1, 0.5 to the GSE6891 application dataset. The number of parameters with PSRF > 1.1 is reported. For each model fit, 3016 parameters were monitored.

*θ*	Number of Parameters
0.01	24
0.1	0
0.5	0

The number of selected features across the values of *θ* are reported in Table 11 for the two selection methods. The BF methods selected similar numbers of features in all cases, and selected fewer features as *θ* increased.

**Table 11:** Number of selected features for BLASSO-FCR model fit with *θ* = 0.01, 0.1, 0.5 to the GSE6891 application dataset. The features were selected using two selection method: *βγ* Bayes factor (BF) greater than 5 or *γ* Bayes factor (BF) greater than 5.

*θ*	*βγ* BF > 5	*γ* BF > 5
0.01	41	41
0.1	7	9
0.5	0	0

Since the *BF*_*γ*_ selection method had good performance in our simulation studies for models fit with *θ* = 0.1, we further explored the selected genomic features. The nine identified probesets and associated genes from the BLASSO-FCR model with *θ* = 0.1 are reported in Table 12. Among these genes, five have been previously associated with leukemia in the literature: *CD109* [48], *GGT5* [49], *PAX8-AS1* [50], *P2RY13* [51], and *UBASH3B* [52].

**Table 12:** Nine probesets identified by BLASSO-FCR models fit with *θ* = 0.1 to the GSE6891 application dataset under censoring (ii). The features were selected using *γ* Bayes factor greater than 5. Genes marked with an asterisk (*) have been previously associated with leukemia.

ID	Gene Symbol
205582_s_at	GGT5*
216950_s_at	*FCGR1A*, *FCGR1B*, *FCGR1C*
220005_at	P2RY13*
226545_at	CD109*
227099_s_at	*C11orf96*
227474_at	PAX8-AS1*
228170_at	*OLIG1*
238587_at	UBASH3B*
239451_at	

## Conclusions

Here we proposed a Bayesian hierarchical FCR model that incorporates the Bayesian LASSO and variable inclusion indicators to identify genomic features that are associated with discrete survival outcomes. These features are candidates for further study that might enhance our understanding of disease and could be potential diagnostic biomarkers and even novel targets for new treatments.

In our simulation study we evaluated the performance of our proposed BLASSO-FCR model using simulated outcomes with five discrete ordinal levels, that used 1000 genomic covariates from the GSE37642 dataset. Thus, the simulated dataset recapitulated the complex relationships among genes in real gene expression data. We evaluated different prior probabilities of inclusion through changing the value of *θ*, different selection methods, and two data settings. We also compared the performance of our BLASSO-FCR model to that of the OGMIFS FCR model.

Generally, our proposed BLASSO-FCR model performed quite well and tended to have higher TPR, TNR, PPV, and NPV and lower FDR compared to the OGMIFS FCR models across the data settings. When using the *γ* BF selection method, the Bayesian models tended to select fewer features compared to the OGMIFS models. We also found there was little difference in the variable selection performance of the Bayesian methods across the data settings.

Despite the good performance, our proposed model has some limitations. In our simulations studies, we limited the number of genomic features to 1000 due to computation time. Further work is needed to speed up this method. We also only used the Bayesian LASSO with variable inclusion indicators in our models, but other regularization priors like the horseshoe could be investigated further [53].

We also applied our proposed BLASSO-FCR model to the GSE6891 gene expression dataset of AML patients less than 60 years old. The models fit with *θ* = 0.1 had no evidence of lack of convergence and selected nine probesets that mapped to ten unique genes. Five of these genes have been previously associated with leukemia.

In conclusion, we proposed a flexible Bayesian FCR model appropriate for discrete survival outcomes. This method allows variable selection and inference to be performed simultaneously and is easy to implement in R. R code demonstrating the usage of the BLASSO-FCR model is available at https://github.com/annaSeffernick/BayesianLassoFCR.

## Supporting information

S1 Appendix Abbreviations, proofs, additional simulation results, and R code. A table of abbreviations used in the manuscript, mathematical proof that the posterior distribution of *β* in the proposed hierarchical model is unimodal, derivation of Bayes Factors, additional simulation design and results, and R code to implement the model.
(PDF)
