# Supplementary material for: Penalized Bayesian forward continuation ratio model with application to high-dimensional data with discrete survival outcomes
Source: PLoS One. 2024 Mar 28;19(3):e0300638. doi: 10.1371/journal.pone.0300638 (PMC10977717; doi:10.1371/journal.pone.0300638)
Supplement: S1 Appendix — A table of abbreviations used in the manuscript, mathematical proof that the posterior distribution of β in the proposed hierarchical model is unimodal, derivation of Bayes Factors, additional simulation design and results, and R code to implement the model. (PDF) [file pone.0300638.s001.pdf]

S1 Appendix: Supplementary materials for “Penalized Bayesian forward continuation ratio model with application to high-dimensional data with discrete survival outcomes”

Anna Eames Seffernick and Kellie J. Archer

January 2024

Contents

1 List of abbreviations **1**

2 Proof of unimodality of the posterior **1**

3 Bayes factor derivation **5**

4 Additional simulation studies **5**

4.1 Additional simulation study results: Oracle value of θ 5

4.2 Additional simulation study results: $\theta = 0.1$ 11

4.3 Additional simulations conclusions: 17

5 R code **20**

1 List of abbreviations

Abbreviations used in the manuscript and supplementary materials are listed alphabetically in Table S1.

2 Proof of unimodality of the posterior

Park and Casella [1] showed that the joint posterior distribution of β and σ^2 under the prior $\Pr(\beta, \sigma^2) = \Pr(\sigma^2) \prod_{m=1}^{P} \frac{\lambda}{2\sqrt{\sigma^2}} e^{-\lambda|\beta_m|/\sqrt{\sigma^2}}$ is unimodal for the hierarchical Bayesian LASSO model for normal data. Zhang [2] proved that the Bayesian LASSO for ordinal models that satisfy the proportional odds assumption leads to a unimodal posterior for β, not conditioning on σ^2, and we proved this for the Bayesian LASSO stereotype model [3].

We prove unimodality of the posterior in a similar manner as for the stereotype model. Assuming censoring occurs at the beginning of the recorded interval, the likelihood is given by

$$L(\mathbf{Y}|\mathbf{x}_i) = \prod_{i=1}^{N} \prod_{k=1}^{K+1} \pi_{ik}^{y_{ik}} (1 - \pi_{ik})^{\sum_{j=k}^{K+1} t_{ij} - y_{ik}}$$

and the log-likelihood is given by

$$\log L(\mathbf{Y}|\mathbf{x})_i) = \sum_{i=1}^{N} \sum_{k=1}^{K+1} \left[y_{ik} \log(\pi_{ik}) + \left(\sum_{j=k}^{K+1} t_{ij} - y_{ik} \right) \log(1 - \pi_{ik}) \right]$$

where t_{ij} is defined as in Equation 7 of the manuscript.

Table S1: **Abbreviations used in the manuscript and supplementary materials listed alphabetically.**

Abbreviation	Definition
AIC	Akaike information criterion
AML	acute myeloid leukemia
BF	Bayes factor
BIC	Bayesian information criterion
BLASSO-FCR	Bayesian LASSO FCR model
CI	credible interval
clog-log	complimentary log-log
ELN	European LeukemiaNet
ESMO	European Society for Medical Oncology
FCR	forward continuation ratio
FDR	false discovery rate
GEO	Gene Expression Omnibus
GMIFS	generalized monotone incremental forward stagewise
HPDI	highest posterior density interval
JAGS	Just Another Gibbs Sampler
LASSO	least absolute shrinkage and selection operator
OGMIFS FCR	frequentist ordinal GMIFS FCR model
OSC	Ohio Supercomputer Center
MCMC	Markov chain Monte Carlo
NPV	negative predictive value
PPV	positive predictive value
$\Pr(\gamma\|D)$	posterior probability of the inclusion indicator
PSRF	potential scale reduction factor
RFS	relapse-free survival
TNR	true negative rate
TPR	true positive rate

Assuming the prior on the regression coefficients $\boldsymbol{\beta}$ is given by

$$f(\boldsymbol{\beta}) = \prod_{m=1}^{P} \frac{\lambda}{2} e^{-\lambda|\beta_m|}$$

we can derive the posterior.

$$\begin{aligned} L(\boldsymbol{\beta}|\mathbf{x}_i, \mathbf{Y}) &\propto L(\mathbf{Y}|\mathbf{x}_i) f(\boldsymbol{\beta}) \\ &\propto \exp(-\lambda||\boldsymbol{\beta}||_1) \prod_{i=1}^{N} \prod_{k=1}^{K+1} \pi_{ik}^{y_{ik}} (1-\pi_{ik})^{\sum_{j=k}^{K+1} t_{ij} - y_{ik}} \end{aligned}$$

and the log-likelihood can be written as

$$\begin{aligned}\log L(\boldsymbol{\beta}|\mathbf{x}_i,\mathbf{Y}) &\propto -\lambda||\boldsymbol{\beta}||_1 + \sum_{i=1}^{N}\sum_{k=1}^{K+1}\left[y_{ik}\log(\pi_{ik}) + \left(\sum_{j=k}^{K+1} t_{ij} - y_{ik}\right)\log(1-\pi_{ik})\right] \\ &\propto -\lambda||\boldsymbol{\beta}||_1 + \sum_{i=1}^{N}\sum_{k=1}^{K+1}[y_{ik}\log(1-\exp\{-\exp(\alpha_k + \mathbf{x}_i^T\boldsymbol{\beta})\}) \\ &+ \left(\sum_{j=k}^{K+1} t_{ij} - y_{ik}\right)\log(\exp\{-\exp(\alpha_k + \mathbf{x}_i^T\boldsymbol{\beta})\})] \\ &\propto -\lambda||\boldsymbol{\beta}||_1 + \sum_{i=1}^{N}\sum_{j=1}^{K+1}[y_{ik}\log(1-\exp\{-\exp(\alpha_k + \mathbf{x}_i^T\boldsymbol{\beta})\}) \\ &+ \left(\sum_{j=k}^{K+1} t_{ij} - y_{ik}\right)(-\exp(\alpha_k + \mathbf{x}_i^T\boldsymbol{\beta}))]\end{aligned}$$

Now we can show that this log-likelihood or the posterior is unimodal with respect to $\boldsymbol{\beta}$. Since $||\boldsymbol{\beta}||_1$ is convex and $-\lambda < 0$, the first term is concave. We can show the remaining terms are concave by taking 2nd derivatives. For $i = 1, ..., N$ and $k = 1, ..., K+1$,

$$\begin{aligned}&\frac{\partial}{\partial \beta_s}\left[y_{ik}\log(1-\exp\{-\exp(\alpha_k + \mathbf{x}_i^T\boldsymbol{\beta})\} + \left(\sum_{j=k}^{K+1} t_{ij} - y_{ik}\right)(-\exp(\alpha_k + \mathbf{x}_i^T\boldsymbol{\beta}))\right] \\ &= \frac{y_{ik}x_{is}\exp\left\{-\exp(\alpha_k + \mathbf{x}_i^T\boldsymbol{\beta})\right\}\exp(\alpha_k + \mathbf{x}_i^T\boldsymbol{\beta})}{1-\exp\left\{-\exp(\alpha_k + \mathbf{x}_i^T\boldsymbol{\beta})\right\}} - \left(\sum_{j=k}^{K+1} t_{ij} - y_{ik}\right)x_{is}\exp(\alpha_k + \mathbf{x}^T\boldsymbol{\beta})\end{aligned}$$

and

$$\begin{aligned}&\frac{\partial^2}{\partial \beta_s^2}\left[y_{ik}\log(1-\exp\{-\exp(\alpha_k + \mathbf{x}_i^T\boldsymbol{\beta})\} + \left(\sum_{j=k}^{K+1} t_{ij} - y_{ik}\right)(-\exp(\alpha_k + \mathbf{x}_i^T\boldsymbol{\beta}))\right] \\ &= \frac{(y_{ik}x_{is}^2\exp\{-\exp(\alpha_k + \mathbf{x}_i^T\boldsymbol{\beta})\}\exp(\alpha_k + \mathbf{x}_i^T\boldsymbol{\beta}))(1-\exp(\alpha_k + \mathbf{x}_i^T\boldsymbol{\beta}) - \exp\{-\exp(\alpha_k + \mathbf{x}_i^T\boldsymbol{\beta})\})}{(1-\exp\{-\exp(\alpha_k + \mathbf{x}_i^T\boldsymbol{\beta})\})^2} \\ &- \left(\sum_{j=k}^{K+1} t_{ij} - y_{ik}\right)x_{is}^2\exp(\alpha_k + \mathbf{x}_i^T\boldsymbol{\beta}) \leq 0\end{aligned}$$

since $(1-\exp(\alpha_k + \mathbf{x}_i^T\boldsymbol{\beta}) - \exp\{-\exp(\alpha_k + \mathbf{x}_i^T\boldsymbol{\beta}\}) \leq 0$ and $\left(\sum_{j=k}^{K+1} t_{ij} - y_{ik}\right)x_{is}^2\exp(\alpha_k + \mathbf{x}_i^T\boldsymbol{\beta}) \geq 0$. Thus, each term in the log-likelihood of the posterior is concave and so the log-likelihood itself is concave. Therefore, the posterior under our proposed Bayesian LASSO FCR model is unimodal with respect to β.

Additional mathematical details: We used the following derivatives in our proof of the unimodality of the posterior.

$$\frac{\partial}{\partial \beta_s}\left[\left(\sum_{j=k}^{K+1} t_{ij} - y_{ik}\right) x_{is}\exp(\alpha_k + \mathbf{x}_i^T\boldsymbol{\beta})\right] = \left(\sum_{j=k}^{K+1} t_{ij} - y_{ik}\right) x_{is}^2\exp(\alpha_k + \mathbf{x}_i^T\boldsymbol{\beta})$$

$$\frac{\partial}{\partial \beta_s}\left[\frac{y_{ik}x_{is}\exp\{-\exp(\alpha_k + \mathbf{x}^T\boldsymbol{\beta})\}\exp(\alpha_k + \mathbf{x}_i^T\boldsymbol{\beta})}{1-\exp\{-\exp(\alpha_k + \mathbf{x}_i^T\boldsymbol{\beta})\}}\right] = \frac{\partial}{\partial \beta_s}\frac{g(\boldsymbol{\beta})}{h(\boldsymbol{\beta})}$$

$$= \frac{g'(\boldsymbol{\beta})h(\boldsymbol{\beta}) - g(\boldsymbol{\beta})h'(\boldsymbol{\beta})}{(h(\boldsymbol{\beta}))^2}$$

$$g(\boldsymbol{\beta}) = y_{ik}x_{is}\exp\{-\exp(\alpha_k + \mathbf{x}_i^T\boldsymbol{\beta})\}\exp(\alpha_k + \mathbf{x}_i^T\boldsymbol{\beta})$$

$$\begin{aligned}g'(\boldsymbol{\beta}) &= y_{ik}x_{is}\exp\{-\exp(\alpha_k + \mathbf{x}_i^T\boldsymbol{\beta})\}(-\exp(\alpha_k + \mathbf{x}_i^T\boldsymbol{\beta}))x_{is}\exp(\alpha_k + \mathbf{x}_i^T\boldsymbol{\beta})\\
&+ y_{ik}x_{is}\exp\{-\exp(\alpha_k + \mathbf{x}_i^T\boldsymbol{\beta})\}\exp(\alpha_k + \mathbf{x}_i^T\boldsymbol{\beta})x_{is}\\
&= -y_{ik}x_{is}^2\exp\{-\exp(\alpha_k + \mathbf{x}_i^T\boldsymbol{\beta})\}[\exp(\alpha_k + \mathbf{x}_i^T\boldsymbol{\beta})]^2\\
&+ y_{ik}x_{is}^2\exp\{-\exp(\alpha_k + \mathbf{x}_i^T\boldsymbol{\beta})\}\exp(\alpha_k + \mathbf{x}_i^T\boldsymbol{\beta})\end{aligned}$$

$$h(\boldsymbol{\beta}) = 1 - \exp\{-\exp(\alpha_k + \mathbf{x}_i^T\boldsymbol{\beta})\}$$

$$\begin{aligned}h'(\boldsymbol{\beta}) &= -\exp\{-\exp(\alpha_k + \mathbf{x}_i^T\boldsymbol{\beta})\}(-\exp(\alpha_k + \mathbf{x}_i^T\boldsymbol{\beta})x_{is})\\
&= x_{is}\exp\{-\exp(\alpha_k + \mathbf{x}_i^T\boldsymbol{\beta})\}\exp(\alpha_k + \mathbf{x}_i^T\boldsymbol{\beta})\end{aligned}$$

$$\begin{aligned}\frac{\partial}{\partial \beta_s}\frac{g(\boldsymbol{\beta})}{h(\boldsymbol{\beta})} &= \frac{[-y_{ik}x_{is}^2\exp\{-\exp(\alpha_k + \mathbf{x}_i^T\boldsymbol{\beta})\}(\exp(\alpha_k + \mathbf{x}_i^T\boldsymbol{\beta}))^2](1-\exp\{-\exp(\alpha_k + \mathbf{x}_i^T\boldsymbol{\beta})\})}{(1-\exp\{-\exp(\alpha_k + \mathbf{x}_i^T\boldsymbol{\beta})\})^2}\\
&+ \frac{[y_{ik}x_{is}^2\exp\{-\exp(\alpha_k + \mathbf{x}_i^T\boldsymbol{\beta})\}\exp(\alpha_k + \mathbf{x}_i^T\boldsymbol{\beta})](1-\exp\{-\exp(\alpha_k + \mathbf{x}_i^T\boldsymbol{\beta})\})}{(1-\exp\{-\exp(\alpha_k + \mathbf{x}_i^T\boldsymbol{\beta})\})^2}\\
&- \frac{y_{ik}x_{is}\exp\{-\exp(\alpha_k + \mathbf{x}_i^T\boldsymbol{\beta})\}\exp(\alpha_k + \mathbf{x}_i^T\boldsymbol{\beta})^2x_{is}\exp\{-\exp(\alpha_k + \mathbf{x}_i^T\boldsymbol{\beta})\}}{(1-\exp\{-\exp(\alpha_k + \mathbf{x}_i^T\boldsymbol{\beta})\})^2}\\
&= -\frac{y_{is}x_{is}^2\exp\{-\exp(\alpha_k + \mathbf{x}_i^T\boldsymbol{\beta})\}(\exp(\alpha_k + \mathbf{x}_i^T\boldsymbol{\beta}))^2}{(1-\exp\{-\exp(\alpha_k + \mathbf{x}_i^T\boldsymbol{\beta})\})^2}\\
&+ \frac{y_{ik}x_{is}^2\exp\{-\exp(\alpha_k + \mathbf{x}_i^T\boldsymbol{\beta})\}\exp(\alpha_k + \mathbf{x}_i^T\boldsymbol{\beta})}{(1-\exp\{-\exp(\alpha_k + \mathbf{x}_i^T\boldsymbol{\beta})\})^2}\\
&+ \frac{y_{ik}x_{is}^2(\exp\{-\exp(\alpha_k + \mathbf{x}_i^T\boldsymbol{\beta})\})^2(\exp(\alpha_k + \mathbf{x}_i^T\boldsymbol{\beta}))^2}{(1-\exp\{-\exp(\alpha_k + \mathbf{x}_i^T\boldsymbol{\beta})\})^2}\\
&- \frac{y_{ik}x_{is}^2(\exp\{-\exp(\alpha_k + \mathbf{x}_i^T\boldsymbol{\beta})\})^2\exp(\alpha_k + \mathbf{x}_i^T\boldsymbol{\beta})}{(1-\exp\{-\exp(\alpha_k + \mathbf{x}_i^T\boldsymbol{\beta})\})^2}\\
&- \frac{y_{ik}x_{is}^2(\exp\{-\exp(\alpha_k + \mathbf{x}_i^T\boldsymbol{\beta})\})^2(\exp(\alpha_k + \mathbf{x}_i^T\boldsymbol{\beta}))^2}{(1-\exp\{-\exp(\alpha_k + \mathbf{x}_i^T\boldsymbol{\beta})\})^2}\\
&= \frac{(y_{ik}x_{is}^2\exp\{-\exp(\alpha_k + \mathbf{x}_i^T\boldsymbol{\beta})\}\exp(\alpha_k + \mathbf{x}_i^T\boldsymbol{\beta}))(1-\exp(\alpha_k + \mathbf{x}_i^T\boldsymbol{\beta}) - \exp\{-\exp(\alpha_k + \mathbf{x}_i^T\boldsymbol{\beta})\})}{(1-\exp\{-\exp(\alpha_k + \mathbf{x}_i^T\boldsymbol{\beta})\})^2}\end{aligned}$$

3 Bayes factor derivation

To find the prior odds when applying BF methodology to β_m or $\beta_m\gamma_m$, we note that the marginal prior for β can be derived as

$$\begin{aligned} f(\beta) &= \int_\lambda f(\beta|\lambda)f(\lambda)d\lambda \\ &= \int_0^\infty \frac{\lambda}{2}\exp(-\lambda|\beta|)\frac{b^a\Gamma(a)}{\lambda}^{a-1}\exp(-b\lambda)d\lambda \\ &= \frac{b^a}{2\Gamma(a)}\int_0^\infty \gamma^a \exp(-\lambda[|\beta|+b])d\lambda \\ &= \frac{b^a\Gamma(a+1)}{2\Gamma(a)}\frac{1}{(|\beta|+b)^{a+1}} \end{aligned}$$

4 Additional simulation studies

We conducted additional simulation studies using the same German AMLCG 1999 clinical trial dataset (GEO accession: GSE37642). We generated unbalanced datasets, varying the sample size ($n = 200, 300, 417$) and the number of truly associated features ($k = 10, 25, 50$) out of 1000 total genomic features. For the full dataset $n = 417$ with $k = 10$ associated features, we additionally generated a dataset with $c = 40\%$ censoring. All datasets were generated as described in the Simulation studies subsection in the Materials and methods section of the main manuscript. Continuous survival times were grouped into five time intervals by splitting every 3 months. Censoring rates for each setting are presented in Table S2. We fit our proposed BLASSO-FCR model as described in the text with the prior inclusion probability θ set to the oracle value (true inclusion probability: 0.01, 0.025, or 0.05), or set to $\theta = 0.1$.

4.1 Additional simulation study results: Oracle value of θ

The convergence results of these additional simulation studies are in Table S3. All settings showed lack of convergence in all simulation replicate datasets, with a range of average percentage of parameters failing to converge across the settings from 2.3% to 5.2%. This lack of convergence may be due to using the oracle values of θ which are quite small. Increasing the number of MCMC iterations or increasing θ could lead to improved convergence performance.

The variable selection performance in terms of FDR, TPR, TNR, PPV, and NPV are reported in Table S4 for all of the simulation scenarios with the oracle prior inclusion probabilities. Figure S1 shows the variable selection performance of the different selection methods for the scenarios with $k = 10$ associated features out of 1000 total features. Variable selection tends to improve as sample size increases for all methods. There appears to be little difference between the original 26% censoring scenario and the case where censoring increased to 40%, denoted by the scenario N417k10c40. The $BF_{\beta\gamma}$ and BF_γ selection methods tend to have higher TPR, and NPV across the sample sizes. These methods have worse TNR, PPV, and FDR compared to the other three selection methods across the sample sizes, thought those methods tended to have many fewer discoveries. We see similar patterns in Figure S2 for the scenarios with $k = 25$ associated features and Figure S3. A comparison of the variable selection results across sample size and number of associated features is shown in Figure S4. The number of discoveries appears to move closer to the truth and FDR decreases as sample size increases. PPV tends to increase as sample size increases.The models with $k = 10$ tend to have better TPR and NPV though NPV is quite high for all scenarios. The TNR is fairly high in all cases and does not appear to have much of a pattern across sample size or number of associated features. The variable selection performance appears somewhat inadequate according to TPR, PPV, and FDR in these simulations. This may be due to the choice of prior probability of inclusion θ, so we explored increasing this from the oracle value in each case to $\theta = 0.1$. A 10% prior probability of inclusion is reasonable in many omics datasets.

Table S2: **Average proportion of samples per time-interval and average interval-specific censoring proportion across the 100 simulated datasets generated using the GSE37642 dataset under multiple settings.**

Setting	Time Interval	Proportion of Samples	Proportion Censored
$n = 200, k = 10$	1	0.405	0.123
	2	0.135	0.241
	3	0.090	0.314
	4	0.060	0.372
	5	0.310	0.508
$n = 200, k = 25$	1	0.479	0.083
	2	0.079	0.405
	3	0.053	0.514
	4	0.042	0.549
	5	0.347	0.761
$n = 200, k = 50$	1	0.433	0.097
	2	0.084	0.434
	3	0.055	0.542
	4	0.044	0.589
	5	0.384	0.767
$n = 300, k = 10$	1	0.367	0.136
	2	0.142	0.249
	3	0.089	0.310
	4	0.066	0.385
	5	0.336	0.532
$n = 300, k = 25$	1	0.429	0.101
	2	0.089	0.388
	3	0.060	0.486
	4	0.046	0.576
	5	0.376	0.781
$n = 300, k = 50$	1	0.464	0.088
	2	0.085	0.362
	3	0.053	0.510
	4	0.044	0.587
	5	0.353	0.764
$n = 417, k = 10, c = 40$	1	0.404	0.186
	2	0.155	0.333
	3	0.097	0.416
	4	0.069	0.444
	5	0.275	0.603
$n = 417, k = 25$	1	0.446	0.100
	2	0.085	0.411
	3	0.055	0.495
	4	0.044	0.595
	5	0.370	0.775
$n = 417, k = 50$	1	0.475	0.090
	2	0.087	0.372
	3	0.054	0.475
	4	0.045	0.559
	5	0.340	0.761

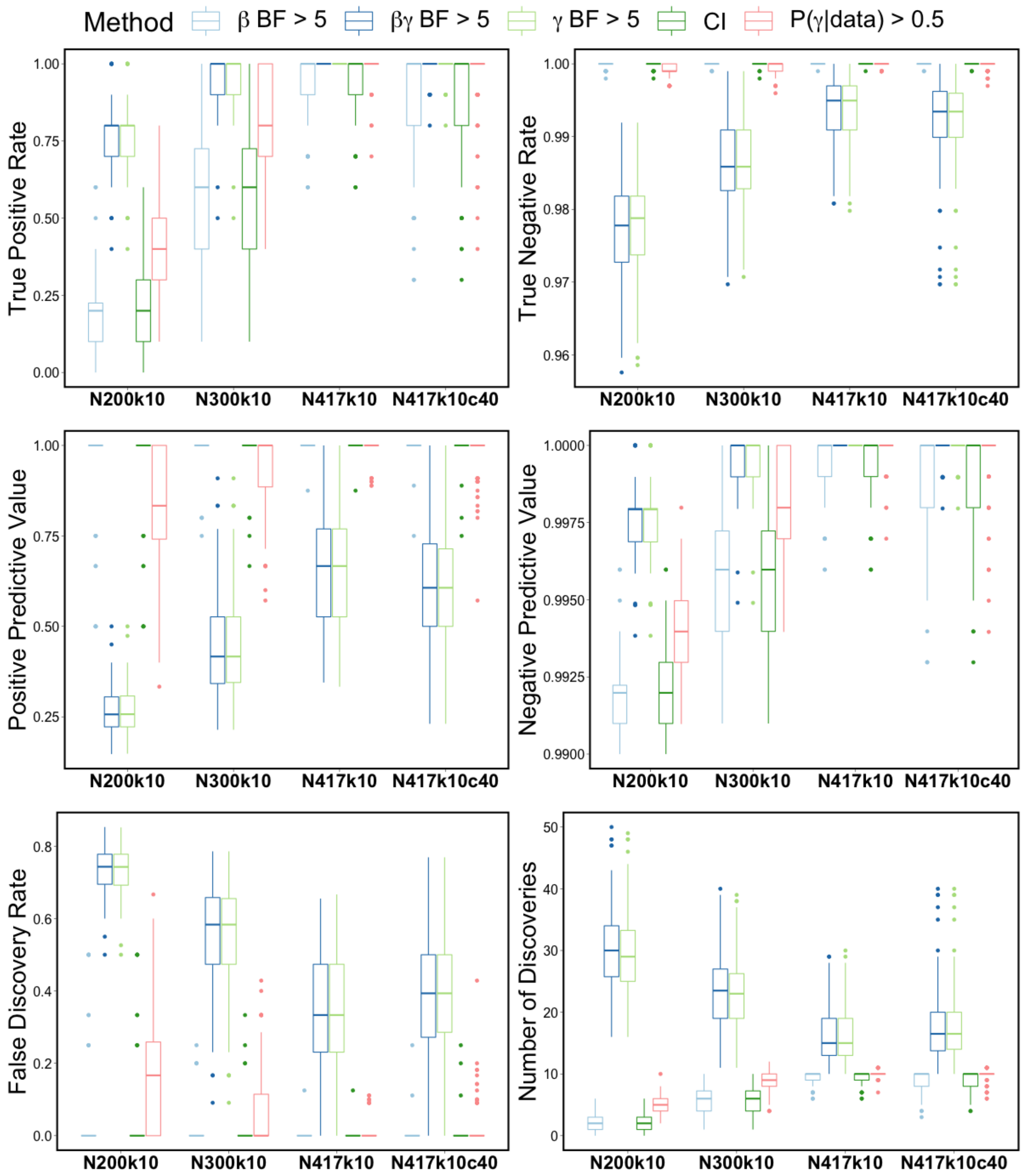

Figure S1: Variable selection performance for Bayesian LASSO FCR model with oracle θ fit to simulated unbalanced data containing 10 truly related features among 1000 covariates from GSE37642 dataset.

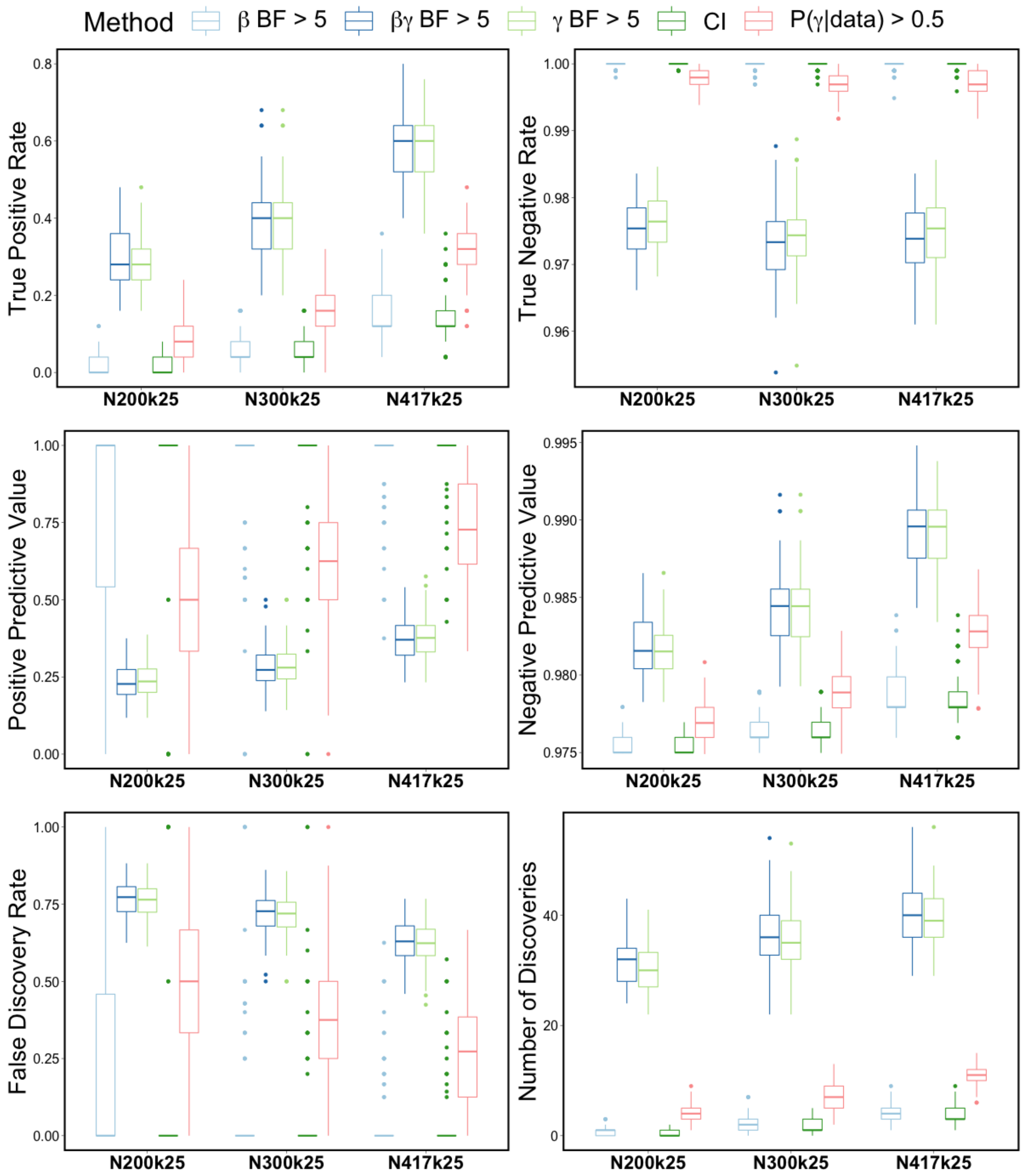

Figure S2: Variable selection performance for Bayesian LASSO FCR model with oracle θ fit to simulated unbalanced data containing 25 truly related features among 1000 covariates from GSE37642 dataset.

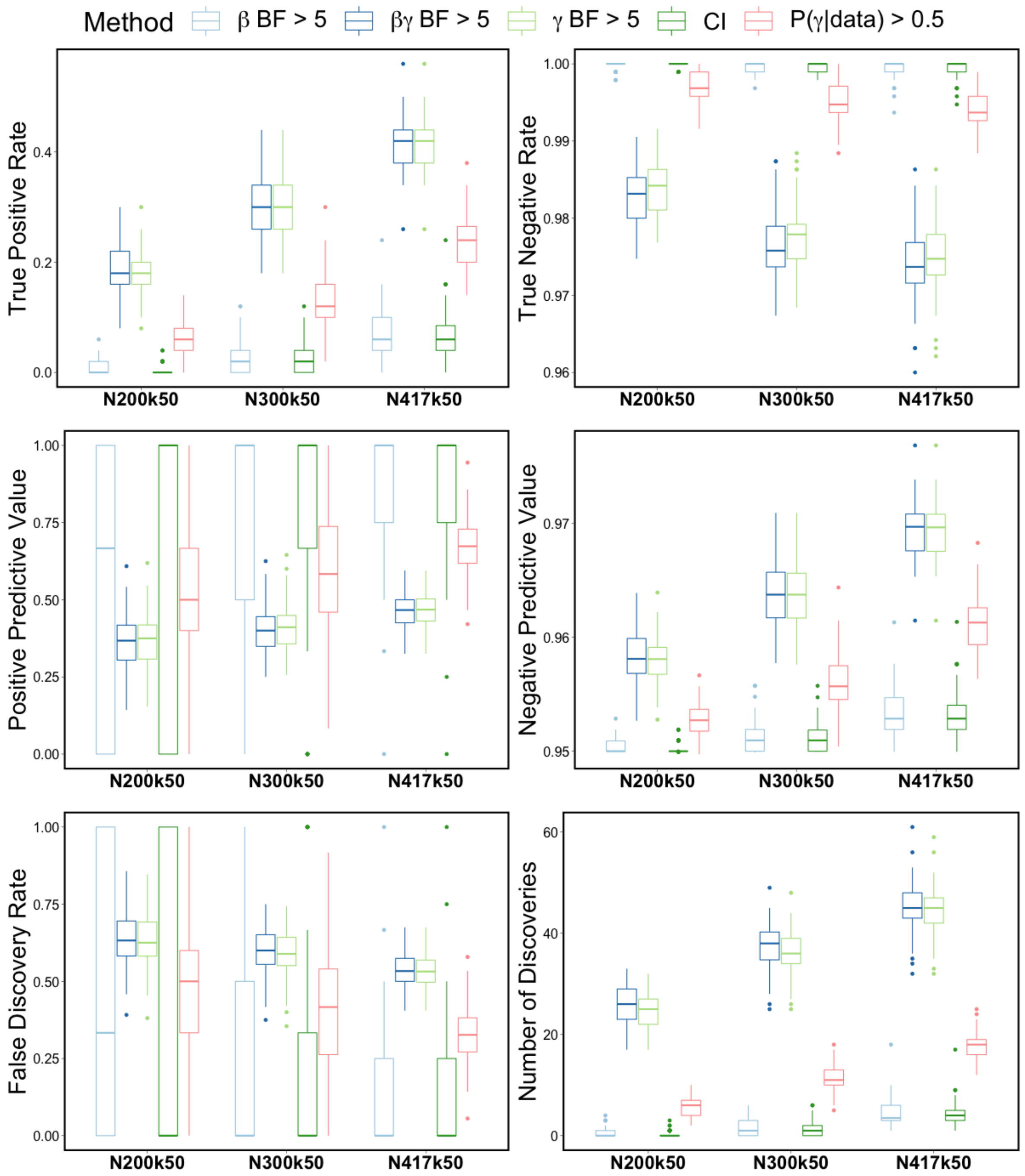

Figure S3: Variable selection performance for Bayesian LASSO FCR model with oracle θ fit to simulated unbalanced data containing 50 truly related features among 1000 covariates from GSE37642 dataset.

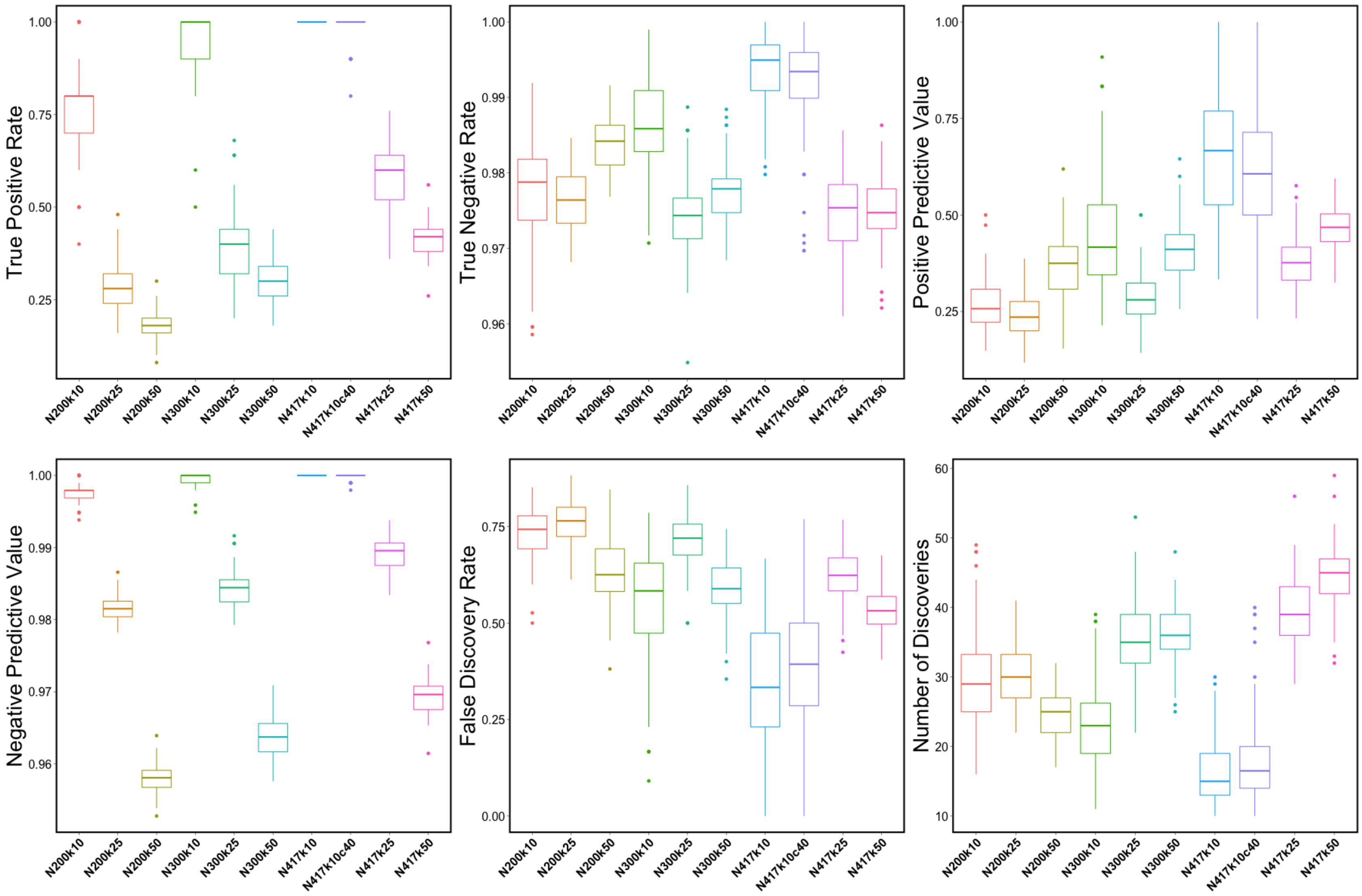

Figure S4: Variable selection performance for Bayesian LASSO FCR model with oracle θ fit to simulated unbalanced data from GSE37642 dataset, with various number of truly related features k and sample sizes n. Variable selection was performed using the BF_γ method.

Table S3: **Lack of convergence in terms of $PSRF > 1.1$ for BLASSO-FCR model fit to additional simulated datasets, with the oracle value of θ. The third column corresponds to the number of datasets out of 100 simulated datasets in which at least one parameter failed to converge. The fourth column corresponds to the average number of parameters out of 3016 monitored parameters that had $PSRF > 1.1$. The * denotes the scenario was reported in main manuscript.**

Setting	θ	Number of Datasets	Mean Number of Parameters
$N = 200, k = 10$	0.01	100	94.77
$N = 200, k = 25$	0.025	100	157.8
$N = 200, k = 50$	0.05	100	126.15
$N = 300, k = 10$	0.01	100	93.32
$N = 300, k = 25$	0.025	100	88.9
$N = 300, k = 50$	0.05	100	107.76
$N = 417, k = 10^*$	0.01	100	129.4
$N = 417, k = 10, c = 40$	0.01	100	119.36
$N = 417, k = 25$	0.025	100	70.03
$N = 417, k = 50$	0.05	100	75.58

4.2 Additional simulation study results: $\theta = 0.1$

The convergence results of these additional simulation studies are in Table S5. The convergence performance has improved compared to the oracle values of θ, with some scenarios containing simulation replicates without any lack of convergence. When there is lack of convergence, the number of affected parameters is much smaller than when the smaller oracle θ values were used in Table S3.

For all scenarios, the variable selection performance in terms of FDR, TPR, TNR, PPV, and NPV are reported in Table S6. In each scenario, the BF_γ and $BF_{\beta\gamma}$ selection methods make many more discoveries than the other selection methods. This makes the results less interpretable, because when most or all of the features are selected, the TPR and PPV will naturally increase. This over-discovery could be due to the choice of BF threshold. When we increase the BF threshold from 5 to 10 we see in Table S7 that the number of discoveries decreases dramatically in all simulation settings. The changes seem most dramatic for the most sparse scenario, when $k = 10$. Increasing the BF threshold for the scenarios with $k = 10$ leads to decreased FDR, slightly lower TPR, much greater TNR, slightly greater PPV, and about the same NPV. Note that FDR is still quite high in these scenarios, and PPV is rather low. Different parameter settings or priors should be explored. At larger values of k, the FDR decreased when BF threshold increased from 5 to 10, but was associated with a much larger drop in TPR than for the $k = 10$ settings.

The variable selection performance for scenarios with 10 truly associated features out of 1000 is shown in Figure S5. As we saw with the oracle values of the prior inclusion probability θ, the performance tends to improve as the sample size increases and there is generally not much of a difference between the original censoring level and the 40% censoring scenario. Figure S6 shows the variable selection perfromance for simulation scenarios with $k = 25$ truly associated features. There is a much more extreme difference between the BF selection methods for γ or $\beta\gamma$ and the other three selection methods in this case. The BF_γ and $BF_{\beta\gamma}$ methods lead to higher TPR and NPV, but lower TNR, PPV, and higher FDR. This is driven by the dramatic increase in the number of discoveries. The results for the $k = 50$ scenarios in Figure S7 show similar patterns, although there is less separation between the methods at each sample size and the BF_γ and $BF_{\beta\gamma}$ methods are selecting closer to the true number of associated features than for the $k = 10$ and $k = 25$ scenarios. Finally, Figure S8 compares all of the scenarios using the $BF_\gamma > 5$ selection method. TPR increases as n increases and decreases as k increases. TNR increases as k increases, very dramatically because there are no or very few "negatives" (unselected features) in the $k = 10$ scenarios. PPV increases both with n and k. The NPV is generally quite high, although missing for the N200k10 scenario because there were no unselected features. FDR decreased as k and n increased.

Table S4: **Average variable selection performance from Bayesian LASSO FCR model with oracle prior inclusion probabilities θ fit to the 100 simulated datasets generated using the GSE37642 dataset under multiple settings. Model was selected using credible intervals (CI), Bayes factors (BF), or mean posterior probability of inclusion ($\Pr(\gamma|D)$). The * denotes the scenario was reported in the main manuscript.**

Setting	Method	Discoveries	FDR	TPR	TNR	PPV	NPV
$n = 200, k = 10$	CI	2.06	0.0371	0.196	0.9999	0.963	0.992
	$\beta\gamma$ BF > 5	30.38	0.737	0.762	0.977	0.263	0.998
	β BF > 5	1.99	0.0375	0.189	0.9999	0.962	0.992
	γ BF > 5	29.8	0.733	0.759	0.978	0.267	0.998
	$\Pr(\gamma|D) > 0.5$	4.86	0.177	0.394	0.999	0.823	0.994
$n = 200, k = 25$	CI	0.45	0.211	0.0144	0.9999	0.789	0.975
	$\beta\gamma$ BF > 5	31.52	0.766	0.293	0.975	0.234	0.982
	β BF > 5	0.74	0.201	0.0232	0.9998	0.799	0.976
	γ BF > 5	30.3	0.759	0.2896	0.976	0.241	0.982
	$\Pr(\gamma|D) > 0.5$	4.16	0.463	0.0872	0.998	0.537	0.977
$n = 200, k = 50$	CI	0.21	0.343	0.0028	0.9999	0.657	0.950
	$\beta\gamma$ BF > 5	25.94	0.635	0.188	0.983	0.365	0.958
	β BF > 5	0.56	0.431	0.0064	0.9997	0.569	0.950
	γ BF > 5	24.8	0.628	0.183	0.984	0.372	0.958
	$\Pr(\gamma|D) > 0.5$	5.73	0.463	0.0592	0.997	0.537	0.953
$n = 300, k = 10$	CI	6.06	0.0098	0.601	0.9999	0.990	0.996
	$\beta\gamma$ BF > 5	23.59	0.558	0.961	0.986	0.442	0.9996
	β BF > 5	5.96	0.0085	0.592	0.99996	0.992	0.996
	γ BF > 5	23.28	0.552	0.961	0.986	0.448	0.9996
	$\Pr(\gamma|D) > 0.5$	8.73	0.072	0.81	0.999	0.928	0.998
$n = 300, k = 25$	CI	1.73	0.092	0.0604	0.9998	0.908	0.976
	$\beta\gamma$ BF > 5	36.31	0.719	0.402	0.973	0.281	0.985
	β BF > 5	1.91	0.122	0.064	0.9997	0.878	0.977
	γ BF > 5	35.28	0.714	0.3972	0.974	0.286	0.984
	$\Pr(\gamma|D) > 0.5$	7.05	0.385	0.169	0.997	0.615	0.979
$n = 300, k = 50$	CI	1.46	0.193	0.0232	0.9997	0.807	0.951
	$\beta\gamma$ BF > 5	37.37	0.593	0.302	0.977	0.407	0.964
	β BF > 5	1.77	0.223	0.0272	0.9996	0.777	0.951
	γ BF > 5	36.42	0.588	0.299	0.977	0.412	0.964
	$\Pr(\gamma|D) > 0.5$	11.32	0.403	0.133	0.995	0.597	0.956
$n = 417, k = 10^*$	CI	9.44	0.00125	0.943	0.99999	0.999	0.999
	$\beta\gamma$ BF > 5	16.25	0.347	1	0.994	0.653	1
	β BF > 5	9.41	0.00125	0.94	0.99999	0.999	0.9994
	γ BF > 5	16.25	0.347	1	0.994	0.653	1
	$\Pr(\gamma|D) > 0.5$	9.98	0.011	0.987	0.9999	0.989	0.9999
$n = 417, k = 10, c = 40$	CI	8.81	0.0056	0.878	0.99997	0.994	0.999
	$\beta\gamma$ BF > 5	17.89	0.392	0.993	0.992	0.608	0.9999
	β BF > 5	8.69	0.0036	0.867	0.99998	0.996	0.999
	γ BF > 5	17.88	0.392	0.993	0.992	0.608	0.9999
	$\Pr(\gamma|D) > 0.5$	9.77	0.025	0.954	0.9998	0.975	0.9995
$n = 417, k = 25$	CI	3.83	0.05042	0.144	0.9998	0.950	0.979
	$\beta\gamma$ BF > 5	40.49	0.631	0.588	0.974	0.369	0.989
	β BF > 5	3.98	0.054	0.149	0.9997	0.946	0.979
	γ BF > 5	39.58	0.625	0.585	0.974	0.375	0.989
	$\Pr(\gamma|D) > 0.5$	10.79	0.271	0.312	0.997	0.729	0.983
$n = 417, k = 50$	CI	3.99	0.119	0.069	0.999	0.881	0.953
	$\beta\gamma$ BF > 5	45.1	0.537	0.416	0.974	0.463	0.969
	β BF > 5	4.24	0.129	0.073	0.999	0.871	0.953
	γ BF > 5	44.4	0.534	0.413	0.975	0.467	0.969
	$\Pr(\gamma|D) > 0.5$	17.55	0.331	0.235	0.994	0.669	0.961

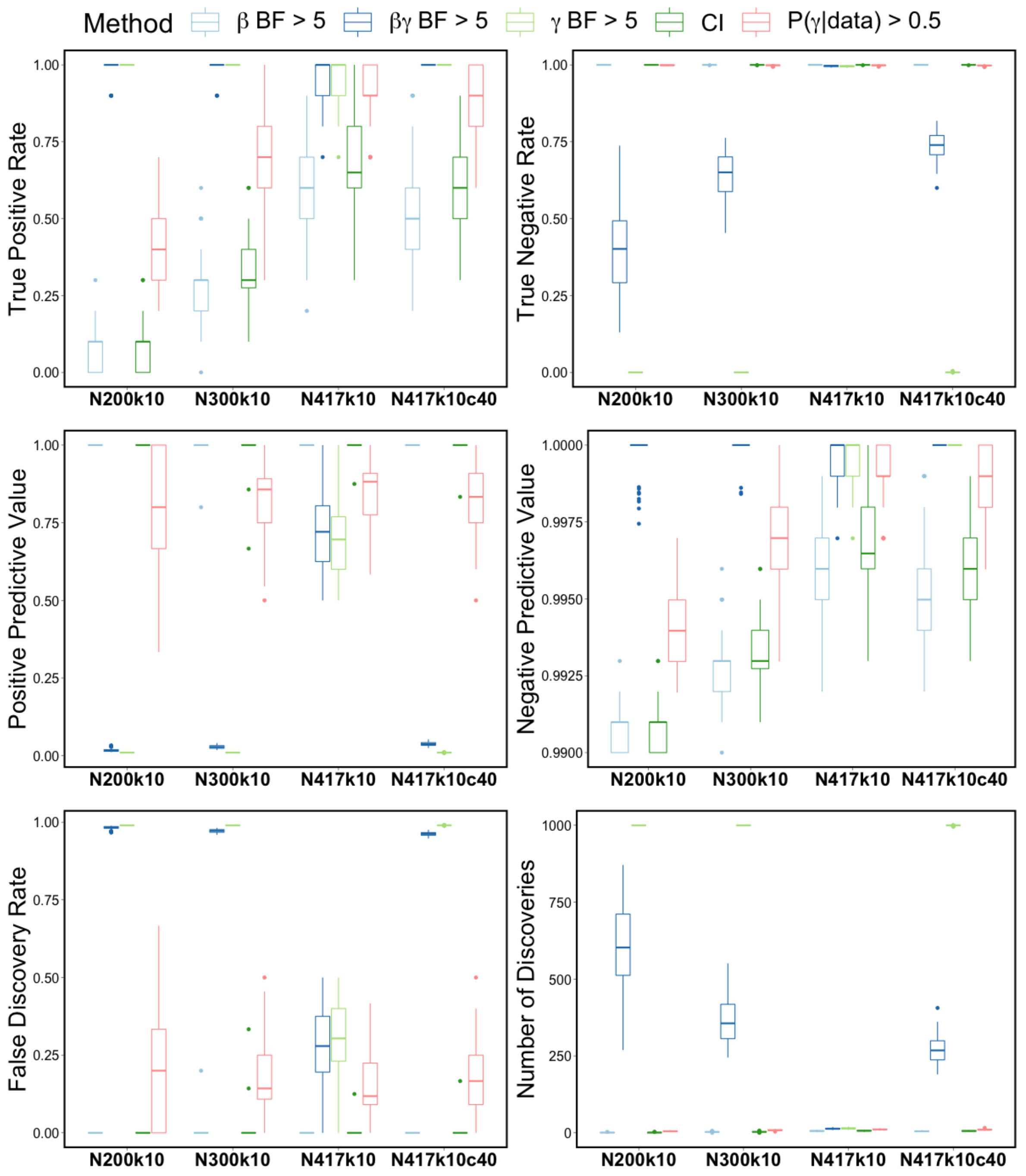

Figure S5: Variable selection performance for Bayesian LASSO FCR model with $\theta = 0.1$ fit to simulated unbalanced data containing 10 truly related features among 1000 covariates from GSE37642 dataset.

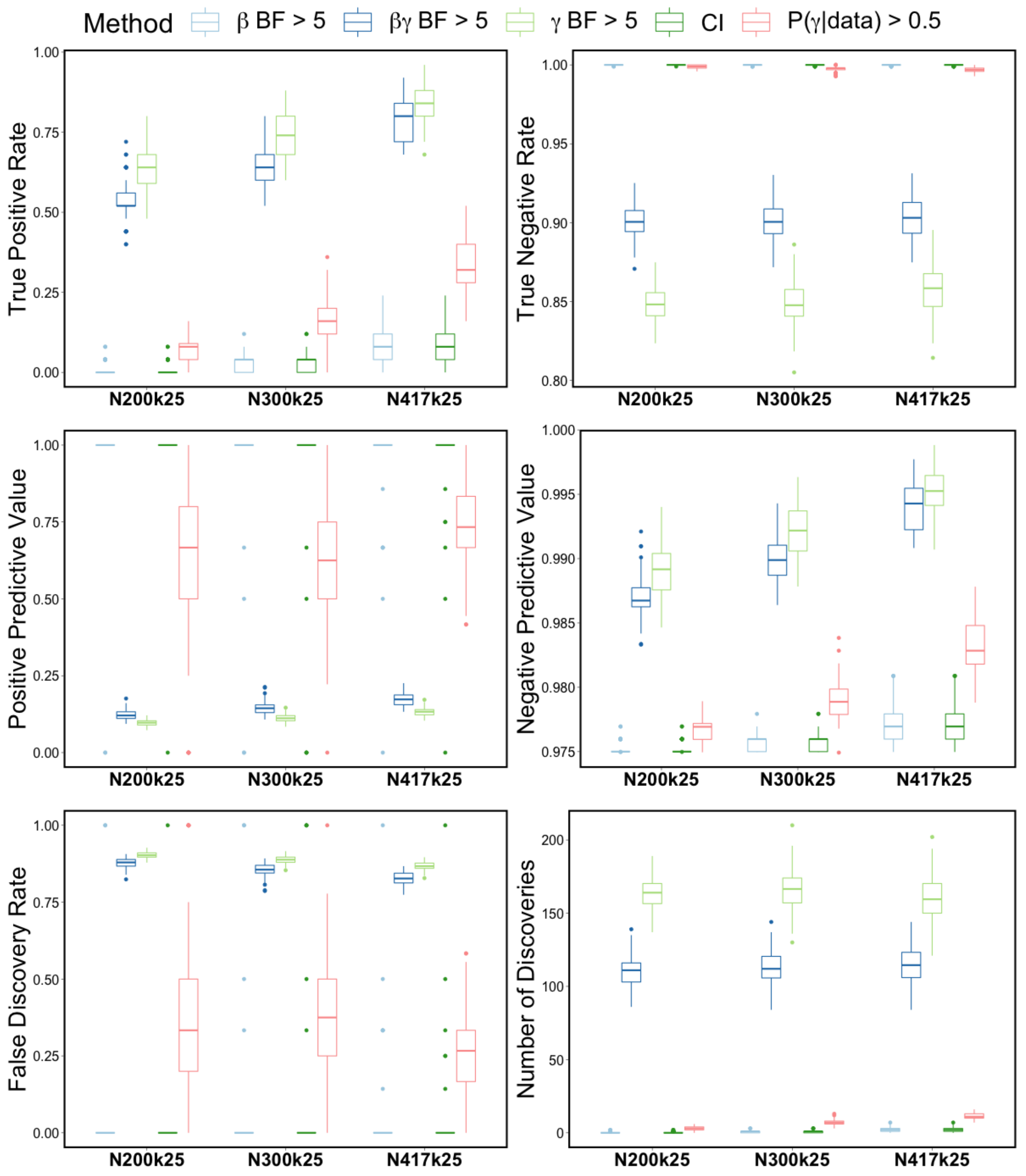

Figure S6: Variable selection performance for Bayesian LASSO FCR model with $\theta = 0.1$ fit to simulated unbalanced data containing 25 truly related features among 1000 covariates from GSE37642 dataset.

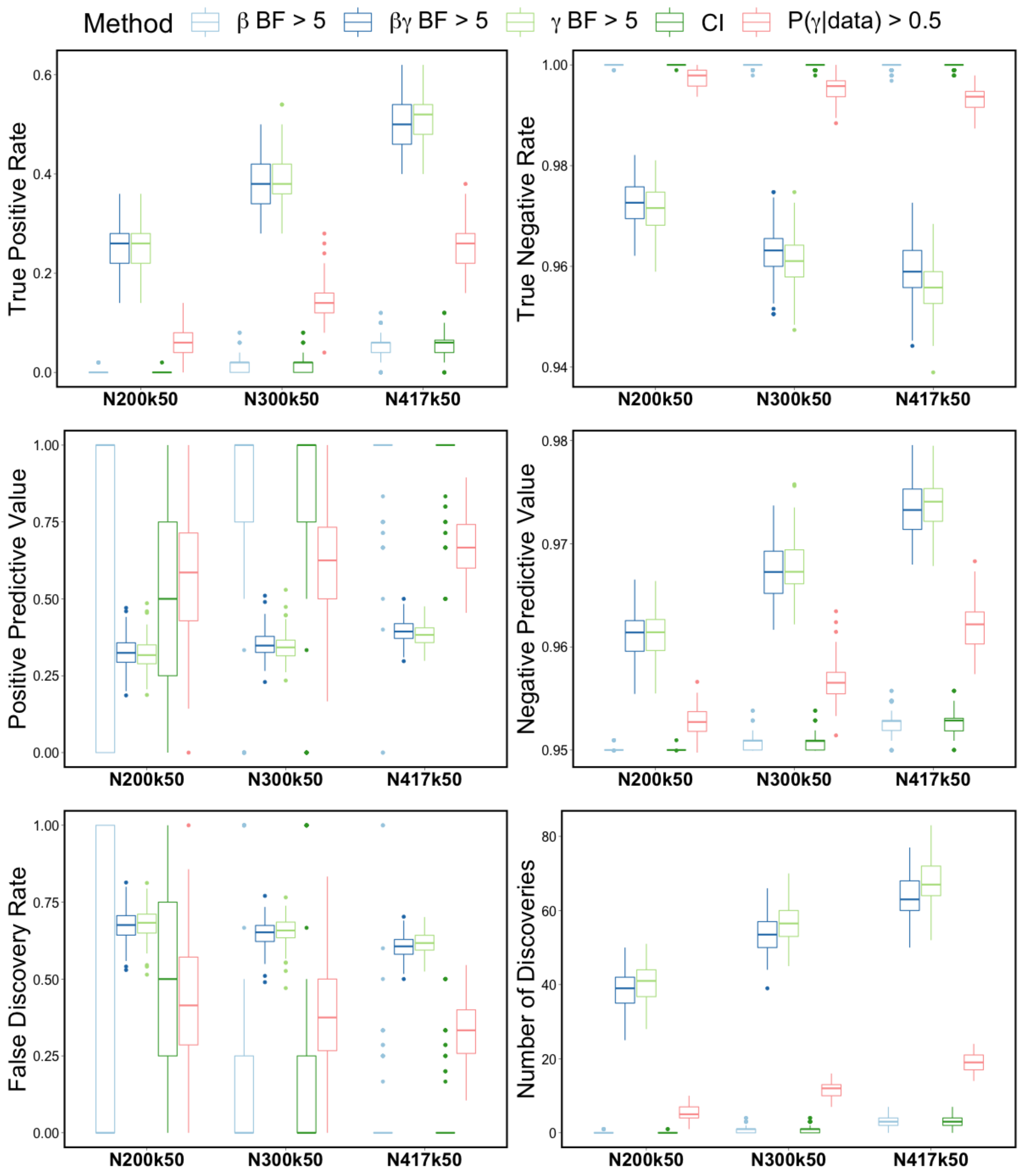

Figure S7: Variable selection performance for Bayesian LASSO FCR model with $\theta = 0.1$ fit to simulated unbalanced data containing 50 truly related features among 1000 covariates from GSE37642 dataset.

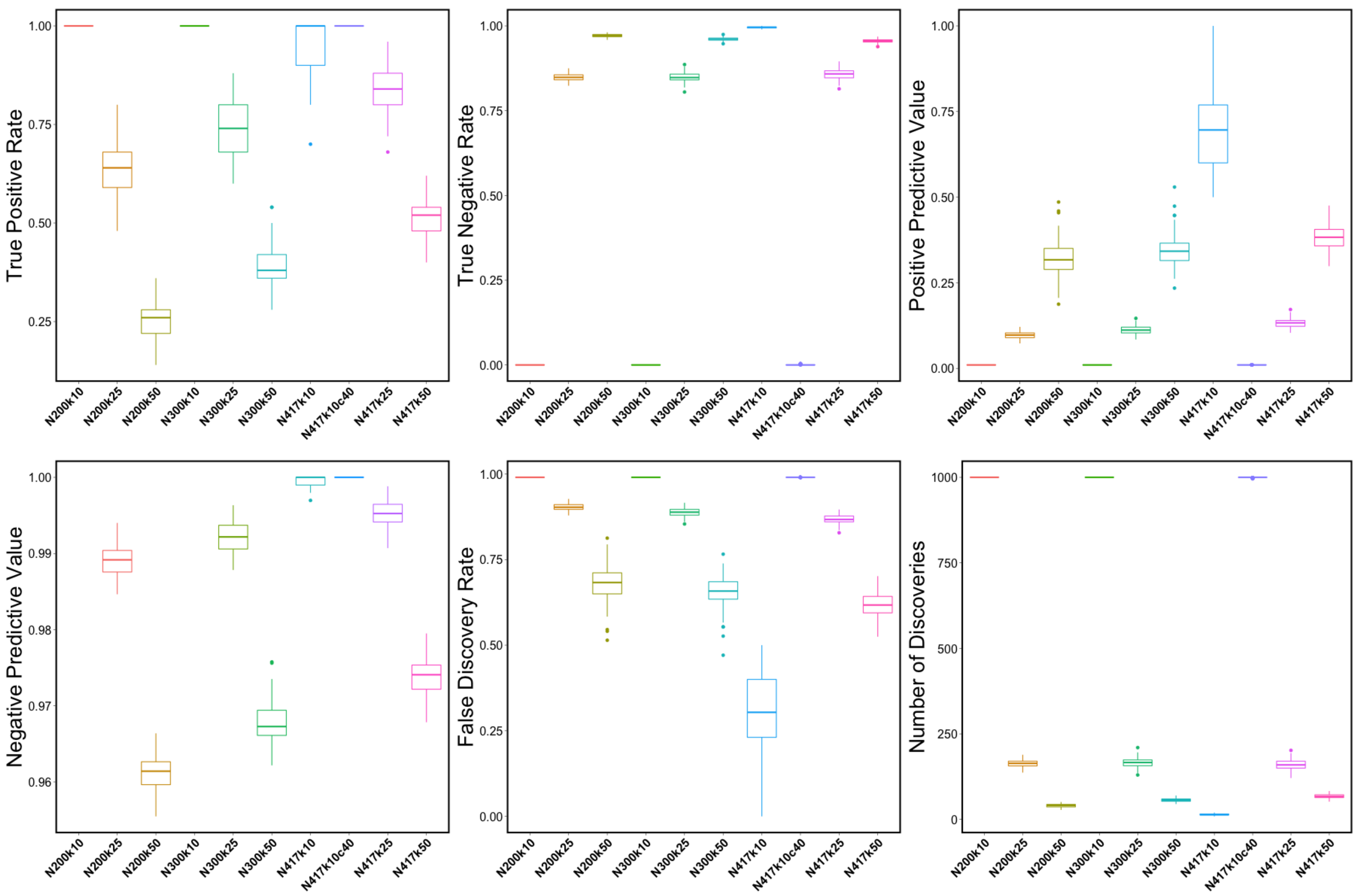

Figure S8: Variable selection performance for Bayesian LASSO FCR model with $\theta = 0.1$ fit to simulated unbalanced data from GSE37642 dataset, with various number of truly related features k and sample sizes n. Variable selection was performed using the BF_γ method.

Table S5: **Lack of convergence in terms of $PSRF > 1.1$ for BLASSO-FCR model fit to additional simulated datasets, with $\theta = 0.1$. The second column corresponds to the number of datasets out of 100 simulated datasets in which at least one parameter failed to converge. The third column corresponds to the average number of parameters out of 3016 monitored parameters that had $PSRF > 1.1$. The * denotes the scenario was reported in main manuscript.**

Setting	Number of Datasets	Mean Number of Parameters
$N = 200, k = 10$	57	2.86
$N = 200, k = 25$	93	6.77
$N = 200, k = 50$	99	26.01
$N = 300, k = 10$	60	1.05
$N = 300, k = 25$	81	4.7
$N = 300, k = 50$	100	19.82
$N = 417, k = 10^*$	81	1.54
$N = 417, k = 10, c = 40$	80	1.43
$N = 417, k = 25$	80	3.32
$N = 417, k = 50$	100	12.42

4.3 Additional simulations conclusions:

As we found in our original simulation studies, the convergence and variable selection performance of the proposed Bayesian LASSO FCR model is sensitive to the choice of the prior inclusion probability, θ. In practice, we often have intuition from subject matter experts about this expected prior probability. We could also place a hyperprior on θ, such as a Beta distribution, which may be more informed by the data. The variable selection performance is also influenced by the choice of Bayes factor threshold. This can be altered in practice to control the number of top/significant hits and can be informed by the scientific questions of interest. In some cases, we may be willing to have a large FDR if we capture all or most of the truly associated features, so we could use a smaller BF threshold to select more features. In other cases, controlling the FDR may be more important and a more stringent (i.e., larger) BF threshold should be used.

Table S6: **Average variable selection performance from Bayesian LASSO FCR model with $\theta = 0.1$ fit to the 100 simulated datasets generated using the GSE37642 dataset under multiple settings. Model was selected using credible intervals (CI), Bayes factors (BF), or mean posterior probability of inclusion ($\Pr(\gamma|D)$). The * denotes the scenario was reported in main manuscript.**

Setting	Method	Discoveries	FDR	TPR	TNR	PPV	NPV
$n = 200, k = 10$	CI	0.8	0	0.08	1	1	0.991
	$\beta\gamma$ BF > 5	607.28	0.983	0.992	0.397	0.0173	0.9999
	β BF > 5	0.69	0	0.069	1	1	0.991
	γ BF > 5	1000	0.99	1	0	0.01	NaN
	$\Pr(\gamma\|D) > 0.5$	5.12	0.220	0.386	0.999	0.780	0.9934
$n = 200, k = 25$	CI	0.2	0.0556	0.0076	0.99999	0.944	0.975
	$\beta\gamma$ BF > 5	110.59	0.877	0.540	0.900	0.123	0.987
	β BF > 5	0.22	0.1	0.008	0.99998	0.9	0.975
	γ BF > 5	163.32	0.903	0.633	0.849	0.097	0.989
	$\Pr(\gamma\|D) > 0.5$	3.05	0.366	0.0764	0.999	0.634	0.977
$n = 200, k = 50$	CI	0.02	0.5	2e-04	0.99999	0.5	0.950
	$\beta\gamma$ BF > 5	38.65	0.672	0.252	0.973	0.328	0.961
	β BF > 5	0.11	0.364	0.0014	0.99996	0.636	0.950
	γ BF > 5	40.3	0.678	0.258	0.971	0.322	0.961
	$\Pr(\gamma\|D) > 0.5$	5.42	0.437	0.0594	0.997	0.563	0.953
$n = 300, k = 10$	CI	3.21	0.00476	0.319	0.99998	0.995	0.993
	$\beta\gamma$ BF > 5	365.58	0.972	0.997	0.641	0.0283	0.99995
	β BF > 5	2.63	0.00202	0.262	0.99999	0.998	0.993
	γ BF > 5	1000	0.99	1	0	0.01	NaN
	$\Pr(\gamma\|D) > 0.5$	8.53	0.171	0.697	0.998	0.829	0.997
$n = 300, k = 25$	CI	0.9	0.0548	0.034	0.99995	0.945	0.976
	$\beta\gamma$ BF > 5	112.28	0.855	0.646	0.901	0.145	0.990
	β BF > 5	0.83	0.0436	0.0316	0.99996	0.956	0.976
	γ BF > 5	165.87	0.888	0.739	0.849	0.112	0.992
	$\Pr(\gamma\|D) > 0.5$	6.97	0.377	0.172	0.997	0.623	0.979
$n = 300, k = 50$	CI	0.88	0.184	0.0144	0.9998	0.816	0.951
	$\beta\gamma$ BF > 5	53.96	0.647	0.380	0.963	0.353	0.967
	β BF > 5	0.93	0.183	0.0152	0.9998	0.817	0.951
	γ BF > 5	56.57	0.654	0.390	0.961	0.346	0.968
	$\Pr(\gamma\|D) > 0.5$	11.7	0.377	0.143	0.995	0.623	0.957
$n = 417, k = 10^*$	CI	6.63	0.00125	0.662	0.99999	0.999	0.997
	$\beta\gamma$ BF > 5	13.52	0.276	0.957	0.996	0.723	0.9996
	β BF > 5	5.74	0	0.574	1	1	0.996
	γ BF > 5	14.25	0.309	0.962	0.995	0.691	0.9996
	$\Pr(\gamma\|D) > 0.5$	10.86	0.146	0.916	0.998	0.854	0.999
$n = 417, k = 10, c = 40$	CI	5.88	0.00167	0.587	0.99999	0.998	0.996
	$\beta\gamma$ BF > 5	269.05	0.962	1	0.738	0.038	1
	β BF > 5	5.02	0	0.502	1	1	0.995
	γ BF > 5	999.56	0.990	1	0.00044	0.010	1
	$\Pr(\gamma\|D) > 0.5$	10.46	0.163	0.865	0.998	0.837	0.999
$n = 417, k = 25$	CI	2.54	0.0255	0.0992	0.99994	0.974	0.977
	$\beta\gamma$ BF > 5	114.53	0.826	0.788	0.903	0.174	0.994
	β BF > 5	2.36	0.0272	0.092	0.9999	0.973	0.977
	γ BF > 5	160.54	0.867	0.847	0.857	0.133	0.995
	$\Pr(\gamma\|D) > 0.5$	11.38	0.265	0.334	0.997	0.735	0.983
$n = 417, k = 50$	CI	2.88	0.0538	0.0536	0.9998	0.946	0.953
	$\beta\gamma$ BF > 5	63.87	0.604	0.503	0.959	0.396	0.973
	β BF > 5	2.82	0.0662	0.052	0.99978	0.934	0.952
	γ BF > 5	67.25	0.617	0.513	0.956	0.383	0.974
	$\Pr(\gamma\|D) > 0.5$	18.85	0.322	0.255	0.994	0.678	0.962

Table S7: **Comparison of average variable selection performance from Bayesian LASSO FCR model with $\theta = 0.1$ fit to the 100 simulated datasets generated using the GSE37642 dataset under multiple settings, with model selection using Bayes factors for γ and $\beta\gamma$ with thresholds of 5 and10.**

Setting	Method	Discoveries	FDR	TPR	TNR	PPV	NPV
$n = 200, k = 10$	$\beta\gamma$ BF > 5	607.28	0.983	0.992	0.397	0.0173	0.9999
	$\beta\gamma$ BF > 10	111.29	0.917	0.899	0.897	0.0832	0.999
	γ BF > 5	1000	0.99	1	0	0.01	NaN
	γ BF > 10	326.09	0.967	0.984	0.681	0.0304	0.9998
$n = 200, k = 25$	$\beta\gamma$ BF > 5	110.59	0.877	0.540	0.900	0.123	0.987
	$\beta\gamma$ BF > 10	26.42	0.736	0.276	0.980	0.264	0.981
	γ BF > 5	163.32	0.903	0.633	0.849	0.097	0.989
	γ BF > 10	29.3	0.752	0.288	0.977	0.248	0.982
$n = 200, k = 50$	$\beta\gamma$ BF > 5	38.65	0.672	0.252	0.973	0.328	0.961
	$\beta\gamma$ BF > 10	13.48	0.539	0.123	0.992	0.461	0.956
	γ BF > 5	40.3	0.678	0.258	0.971	0.322	0.961
	γ BF > 10	13.21	0.535	0.122	0.992	0.465	0.955
$n = 300, k = 10$	$\beta\gamma$ BF > 5	365.58	0.972	0.997	0.641	0.0283	0.99995
	$\beta\gamma$ BF > 10	97.03	0.896	0.977	0.912	0.104	0.9997
	γ BF > 5	1000	0.99	1	0	0.01	NaN
	γ BF > 10	257.41	0.961	0.993	0.750	0.039	0.99991
$n = 300, k = 25$	$\beta\gamma$ BF > 5	112.28	0.855	0.646	0.901	0.145	0.990
	$\beta\gamma$ BF > 10	36.28	0.712	0.415	0.973	0.288	0.985
	γ BF > 5	165.87	0.888	0.739	0.849	0.112	0.992
	γ BF > 10	40.72	0.729	0.439	0.969	0.271	0.985
$n = 300, k = 50$	$\beta\gamma$ BF > 5	53.96	0.647	0.380	0.963	0.353	0.967
	$\beta\gamma$ BF > 10	23.66	0.501	0.234	0.987	0.499	0.961
	γ BF > 5	56.57	0.654	0.390	0.961	0.346	0.968
	γ BF > 10	23.5	0.501	0.233	0.988	0.499	0.961
$n = 417, k = 10, c = 40$	$\beta\gamma$ BF > 5	269.05	0.962	1	0.738	0.038	1
	$\beta\gamma$ BF > 10	84.76	0.879	0.993	0.924	0.121	0.99992
	γ BF > 5	999.56	0.990	1	0.00044	0.010	1
	γ BF > 10	219.26	0.954	1	0.789	0.046	1
$n = 417, k = 25$	$\beta\gamma$ BF > 5	114.53	0.826	0.788	0.903	0.174	0.994
	$\beta\gamma$ BF > 10	42.9	0.644	0.605	0.972	0.356	0.990
	γ BF > 5	160.54	0.867	0.847	0.857	0.133	0.995
	γ BF > 10	47.35	0.666	0.626	0.967	0.334	0.990
$n = 417, k = 50$	$\beta\gamma$ BF > 5	63.87	0.604	0.503	0.959	0.396	0.973
	$\beta\gamma$ BF > 10	32.3	0.446	0.356	0.985	0.554	0.967
	γ BF > 5	67.25	0.617	0.513	0.956	0.383	0.974
	γ BF > 10	32.3	0.447	0.356	0.985	0.553	0.967

5 R code

```
rm(list=ls())
library(runjags)
library(VGAM)
library(parallel)
library(tidyr)
library(GEOquery)

seed1.tmp <- 2204
seed2.tmp <- 1229


# Load data
load("GSE37642_Sim_1000_2.RData")


## Restructure the data

temp.data.m <- sim.list[[1]]
Xmat <- as.matrix(temp.data.m[,-c(1,2,3)])
temp.data <- as.data.frame(temp.data.m)
y <- temp.data$OS5
relapsed <- temp.data$censor

levels <- sort(unique(y))
K <- length(unique(y))
Ymat <- matrix(0, nrow=length(y), ncol=K)

c.y <- ifelse(relapsed == 1, 0, 1) * y
# y.event = time point subj experienced the event;
# y.event = 0 if subj was censored
y.event <- y * relapsed
Tmat <- matrix(0, nrow=length(y), ncol=K)
for(j in levels){
  Ymat[which(y.event == j), which(levels == j)] <- 1
  Tmat[which(y.event == j), which(levels == j)] <- 1
  if(j!=K) {
    Tmat[which(c.y == (j + 1)), which(levels == j)] <- 1
  }
}
YColsum <- colSums(Ymat)

alpha.vec <- numeric()
pi.0 <- table(y)/length(y)
tab <- table(y)

Cum.Tmat <- matrix(0, nrow=nrow(Tmat), ncol=K)
for(i in 1:(K-1)) {
  alpha.vec[i] <- log(-log(1 - (tab[i] / sum(tab[i:K]))))
  Cum.Tmat[, i] <- rowSums(Tmat[, i:K])
}
Cum.Tmat[, K] <- Tmat[, K]
alpha.vec[K] <- log(-log(1 - .99))

data1_wide <- cbind.data.frame(Ymat, Xmat)
colnames(data1_wide)[1:5] <- c("Y1", "Y2", "Y3", "Y4", "Y5")
```

```
dontchange <- colnames(data1_wide)[6:length(colnames(data1_wide))]
data1_long <- gather(data1_wide, key="Y", value="Succ",-all_of(dontchange))
data1_wide_T <- cbind.data.frame(Cum.Tmat, Xmat)
colnames(data1_wide_T)[1:5] <- c("CumT1", "CumT2", "CumT3", "CumT4", "CumT5")
dontchange2 <- colnames(data1_wide_T)[6:length(colnames(data1_wide_T))]
data1_long_T <- gather(data1_wide_T, key="CumT", value="Trials",
                       -all_of(dontchange2))
data1_long_comb <- cbind.data.frame(data1_long, data1_long_T[,grep("Trials",
                                    colnames(data1_long_T))])
data_long_cens1 <- data1_long_comb
colnames(data_long_cens1)[length(colnames(data_long_cens1))] <- "Trials"
data_long_cens1_sm <- data_long_cens1[-which(data_long_cens1$Trials==0),]
data_long_cens1_sm$cp1 <- ifelse(data_long_cens1_sm$Y=="Y1", 1, 0)
data_long_cens1_sm$cp2 <- ifelse(data_long_cens1_sm$Y=="Y2", 1, 0)
data_long_cens1_sm$cp3 <- ifelse(data_long_cens1_sm$Y=="Y3", 1, 0)
data_long_cens1_sm$cp4 <- ifelse(data_long_cens1_sm$Y=="Y4", 1, 0)
data_long_cens1_sm$cp5 <- ifelse(data_long_cens1_sm$Y=="Y5", 1, 0)


# Write the model file
Model = "model{
  for(i in 1:N){
    mu[i] <- inprod(betgma[], X[i,])
    pi[i] <- icloglog(mu[i])
    Y[i] ~ dbern(pi[i])
  }
  for(l in 1:K){
    beta0[l] ~ dnorm(0, 0.1)
  }
  beta[1:K] <- sort(beta0[1:K])
  for(k in (K+1):P){
     beta[k] ~ ddexp(0, lambda)
  }
  lambda ~ dgamma(0.1, 0.1)
  for(s in 1:K){
    gamma[s] <- 1
    betgma[s] <- beta[s]*gamma[s]
  }
  for(m in (K+1):P){
    betgma[m] <- beta[m]*gamma[m]
    gamma[m] ~ dbern(0.01)
  }

  #inits# beta, lambda, .RNG.seed, .RNG.name
  #monitor# beta, lambda, gamma, betgma
  #modules# glm on
}"


JAGSFILE="BLassoDisSurv.bug"
cat(Model, file=JAGSFILE)

##Set Model Parameters
Xmat <- data_long_cens1_sm[,c(grep("cp", colnames(data_long_cens1_sm)),
                              grep("at", colnames(data_long_cens1_sm)))]
N <- dim(data_long_cens1_sm)[1]
```

```
P <- dim(Xmat)[2]

#Data List
dataList <- list("Y" = data_long_cens1_sm$Succ, "X" = as.matrix(Xmat),
                 "N" = N, "P" = P, "K"=K)
# Parameters to be monitored
parameters <- c("beta", "gamma", "lambda", "betgma")

seed1 <- seed1.tmp + 1
set.seed(seed1)
inits1 <- list("beta0" = alpha.vec,"beta" = c(NA, NA, NA, NA, NA,
                rep(0.0, P-5)), "lambda" = rgamma(1, shape=0.1, rate=0.1))
inits2 <- list("beta0" = alpha.vec+0.2, "beta" = c(NA, NA,NA, NA, NA,
                rep(0.0, P-5)), "lambda" = rgamma(1, shape=0.1, rate=0.1))
inits3 <- list("beta0" = alpha.vec+0.05, "beta" = c(NA, NA,NA, NA, NA,
                rep(0.0, P-5)), "lambda" = rgamma(1, shape=0.1, rate=0.1))
inits.list <- list(inits1, inits2, inits3)
names(inits.list) <- c("chain1", "chain2", "chain3")

.RNG.seed <- function(chain){
    return(switch(chain, "1" = seed1+1, "2" = seed1+2, "3"=seed1+3))
}

.RNG.name <- function(chain){
    return(switch(chain, "1" = "base::Super-Duper",
    "2" = "base::Wichmann-Hill", "3"="base::Super-Duper"))
}

library(parallel)
cl <- makeCluster(10)
seed2 <- seed2.tmp + 1
set.seed(seed2)
model.fit.parallel.post.BLI <- run.jags(model=Model, data=dataList,
                                        n.chains=3, inits=inits.list,
                                        burnin=500, adapt=500, sample=3333,
                                        thin=3, method="parallel", cl=cl)
stopCluster(cl)
# Save
save.image("GSE37642P10BLassoScenario2_Point01_Dataset1.RData")
```

References

[1] Trevor Park and George Casella. The Bayesian lasso. *Journal of the American Statistical Association*, 103(482):681–686, 2008.

[2] Yiran Zhang. *Bayesian Variable Selection for High-Dimensional Data with an Ordinal Response*. The Ohio State University, 2019.

[3] Anna Eames Seffernick, Krzysztof Mrózek, Deedra Nicolet, Richard M Stone, Ann-Kathrin Eisfeld, John C Byrd, and Kellie J Archer. High-dimensional genomic feature selection with the ordered stereotype logit model. *Briefings in Bioinformatics*, 23(6):bbac414, 2022.
